# Narcissism and Wellbeing: A Cross-Cultural Meta-Analysis

**DOI:** 10.1177/01461672241307531

**Published:** 2024-12-31

**Authors:** Constantine Sedikides, Yixin Tang, Yan Liu, Eva de Boer, Mark Assink, Sander Thomaes, Eddie Brummelman

**Affiliations:** 1 University of Southampton, UK; 2 Utrecht University, The Netherlands; 3 University of Amsterdam, The Netherlands

**Keywords:** narcissism, hedonic wellbeing, eudaimonic wellbeing, self-esteem, individualism

## Abstract

Do narcissists enjoy better or worse wellbeing than others? Psychological theories disagree. In an attempt to reconcile them, we conducted a comprehensive cross-cultural meta-analysis testing the core hypotheses that grandiose narcissism would be associated with better wellbeing and vulnerable narcissism with worse wellbeing. We also hypothesized that these associations would be explained by self-esteem and would be stronger in countries higher on individualism. First, as hypothesized, grandiose narcissism was associated with better wellbeing and vulnerable narcissism with worse wellbeing. Second, as hypothesized, both associations became nonsignificant after controlling for self-esteem, suggesting that they are explained by self-esteem. Third, partly as hypothesized, the association between grandiose—but not vulnerable—narcissism and wellbeing was stronger in more individualistic countries. Results held across wellbeing forms (hedonic, eudaimonic) and methods (cross-sectional, longitudinal). Advancing psychological theory, we demonstrated that only grandiose narcissists enjoy better wellbeing, especially in individualistic countries, a phenomenon accounted for by their higher self-esteem.

The personality trait of narcissism continues to fascinate scholars and the public. Psychologists have long speculated about its subjective benefits: Is being narcissistic advantageous to one’s wellbeing? We addressed this question in a comprehensive cross-cultural meta-analysis, including both cross-sectional and longitudinal data.

Some perspectives suggest that narcissism is largely harmful to wellbeing (Kernberg, 1975; Ronningstam, 2005), whereas others suggest that narcissism is largely beneficial to it (Blasco-Belled et al., 2024; Zuckerman & O’Loughlin, 2009). We took two critical steps to reconcile these discrepant perspectives. First, we distinguished between grandiose narcissism (marked by relatively high self-esteem) and vulnerable narcissism (marked by relatively low self-esteem). Second, we explored whether the narcissism–wellbeing relation varies across cultures and tested whether this relation is moderated by country-level individualism. In doing so, our work makes novel theoretical contributions to understanding the subjective benefits of narcissism.

We hypothesized that grandiose narcissism would be *positively* related to wellbeing, whereas vulnerable narcissism would be *negatively* related to it. We formulated two follow-up hypotheses. Specifically, we hypothesized that the associations of grandiose and vulnerable narcissism with wellbeing would be (a) explained by self-esteem and (b) stronger in countries higher in individualism.

## Hedonic and Eudaimonic Wellbeing

Our meta-analysis adopts a broad conceptualization of wellbeing, which has two components. *Hedonic (or subjective) wellbeing* is imbued with positive emotionality. It refers to the extent to which individuals experience positive affect and judge their lives as being satisfying (Killingsworth et al., 2023; Layous et al., 2014). That is, hedonic wellbeing has an affective component (the “hallmark” of happiness; Layous & Lyubomirsky, 2014, p. 473) and a cognitive component (Emerson et al., 2017; Pavot & Diener, 2008).^1^ *Eudaimonic (or psychological) wellbeing* is more purposeful (Sheldon, 2018) and complex (Vittersø, 2016). It can comprise subjective vitality, life meaningfulness, autonomy, personal growth, optimism, spirituality, positive relationships, and competence or environmental mastery (Ryff, 1989; Su et al., 2014). Although hedonic and eudaimonic wellbeing often go hand in hand (Disabato et al., 2016; Kashdan et al., 2008), they can diverge (Huta & Waterman, 2014; Joshanloo, 2016). When they do so, hedonic wellbeing captures “feeling good” or judging one’s life as satisfactory, whereas eudaimonic wellbeing likely captures “doing good” (e.g., having meaning in life, positive relationships, or a sense of accomplishment; Sheldon, 2018).

## Grandiose Versus Vulnerable Narcissism

Do narcissists enjoy better or worse wellbeing than others? That might depend on the form of narcissism: grandiose or vulnerable (Sedikides, 2021; Thomaes et al., 2018). These are either unrelated or weakly and positively related (Jauk et al., 2017; Miller et al., 2011). According to recent theorizing, they share a common core of entitlement and self-importance (Krizan & Herlache, 2018) or interpersonal antagonism (Miller et al., 2016, 2017). Although sharing this core, grandiose narcissism is more approach oriented and marked by extraversion, whereas vulnerable narcissism is more avoidance oriented and marked by neuroticism.

*Grandiose narcissists* are characterized by self-confidence, extraversion, optimism, exhibitionism, dominance, manipulativeness, and risk seeking (Sedikides, 2021; Thomaes et al., 2018). They self-enhance (i.e., have inflated self-views) on the agentic domain, such as intelligence, creativity, and vision, and they can be admirative or rivalrous. They also pursue and maintain their inflated self-views by seeking admiration via assertive self-promotion (Back et al., 2013; Grapsas et al., 2020). Very few studies addressed specific relations between admirative or rivalrous narcissism, on the one hand, and wellbeing, on the other, and so we collapsed across forms of grandiose narcissism.^2^ *Vulnerable narcissists* are characterized by introversion, neuroticism, pessimism, withdrawal, and a defensive or reactive interpersonal orientation (Du et al., 2022; Miller et al., 2018). Unlike grandiose narcissists, they do not self-enhance on the agentic domain (Brown et al., 2016).

We hypothesized a positive relation between grandiose narcissism and wellbeing. There is little evidence that grandiose narcissists suffer from internal turmoil (Thomaes et al., 2018). Instead, they are adept at emotion regulation and manifest a positive socioemotional orientation (e.g., reward sensitivity, boldness; Czarna et al., 2018; Zhang et al., 2017). In contrast, we hypothesized a negative relation between vulnerable narcissism and wellbeing. The evidence indicates that vulnerable narcissists have a turbulent internal life (Krizan & Herlache, 2018; Miller et al., 2018). They are characterized by emotion dysregulation and a negative socioemotional orientation (e.g., predisposition to experience shame or envy; Czarna et al., 2018; Freis et al., 2015). Furthermore, we explored the possibility that the presumed associations of grandiose and vulnerable narcissism with wellbeing generalize across hedonic and eudaimonic wellbeing.

## Self-Esteem as Mechanism

What explains the association of grandiose and vulnerable narcissism with wellbeing? One possible mechanism is self-esteem. *Self-esteem* refers to an explicit and global evaluation of the self (Sedikides & Gregg, 2003), that is, one’s sense of worth as a person (Rosenberg, 1965). Self-esteem prospectively predicts both hedonic and eudaimonic wellbeing (Orth & Robins, 2014, 2022). For example, individuals with high self-esteem are better able to bounce back from setbacks, form more satisfying social relationships, and are more optimistic and resilient in their goal pursuits (Leary & Baumeister, 2000). Grandiose narcissism is characterized by high self-esteem. Although grandiose narcissism has long been defined as a form of high self-esteem, recent research shows they are distinct (Brummelman et al., 2016, 2018; Thomaes & Brummelman, 2016). By contrast, vulnerable narcissism is characterized by low self-esteem. Thus, we hypothesized that the greater wellbeing of grandiose narcissists is explained by their high self-esteem, whereas the worse wellbeing of vulnerable narcissists is explained by their low self-esteem.

## Person–Culture Fit

Narcissism might have different implications for wellbeing, depending on salient cultural values of the society in which one lives. *Individualism* refers to being self-contained or self-reliant, and to placing greater importance on personal achievements or individual rights (Hofstede & Bond, 1984; Triandis, 2001). We were particularly interested in country-level individualism: the cumulative degree of independence among members of society (Hofstede et al., 2010).

Person–environment fit research indicates that culture can moderate associations at the personality level, consistent with person–environment fit perspectives (Gebauer et al., 2013, 2017; Van Vianen, 2018). Based on this person–environment fit perspective, we theorized that individualism would moderate the association of narcissism with wellbeing. Cultures high on individualism tend to value agency (e.g., self-mastery, status victory, achievement responsibility, empowerment; McAdams, 2001). Consistent with their agentic (e.g., extraverted, approach-oriented) nature, grandiose narcissists in individualistic cultures may experience fit between the kind of person they are and the kind of person their culture expects them to be, potentially contributing to their wellbeing. By contrast, consistent with their nonagentic (e.g., introverted, avoidance oriented) nature, vulnerable narcissists in individualistic cultures may experience a lack of fit between the kind of person they are and the kind of person their culture expects them to be, potentially undermining their wellbeing. Thus, in more individualistic cultures, grandiose and vulnerable narcissists may experience diverging wellbeing. Accordingly, grandiose narcissism may show a better fit with countries high in individualism, rendering it more conducive to wellbeing, whereas vulnerable narcissism may show a better fit with countries low in individualism, rendering it less detrimental to wellbeing. Thus, we hypothesized that individualism would moderate the positive association between grandiose narcissism and wellbeing, with this relation being stronger (i.e., increasingly positive) in more individualistic cultures. Furthermore, we hypothesized that individualism would moderate the negative association between vulnerable narcissism and wellbeing, with this relation being stronger (i.e., increasingly negative) in more individualistic cultures.

## Existing Meta-Analyses

Two meta-analyses considered the association between narcissism and wellbeing. One reported a small positive relation between grandiose narcissism and a personal adjustment index comprising life satisfaction, positive affect, negative affect, and depression (Dufner et al., 2019). Another meta-analysis, focusing on The Dark Triad (psychopathy, Machiavellianism, narcissism) and wellbeing, examined grandiose and vulnerable narcissism in relation to aspects of both hedonic wellbeing^3^ (positive affect, happiness, life satisfaction, negative affect) and eudaimonic wellbeing (self-acceptance, autonomy, personal growth, positive relationships, purpose in life, environmental mastery, flourishing; Blasco-Belled et al., 2024). It found a significant positive association of grandiose narcissism, and a significant negative association of vulnerable narcissism, with subjective wellbeing. Furthermore, it found a nonsignificant association of grandiose narcissism, and a significant negative association of vulnerable narcissism, with eudaimonic wellbeing.

Collectively, these meta-analyses have limitations. First, they were rather narrow in scope, featuring a small number of effect sizes. Second, they did not adopt a cross-cultural perspective, and were thus unable to examine person–culture fit. Third, they did not test whether self-esteem explained the associations of narcissism with wellbeing. We addressed these limitations here. Our meta-analysis, then, is (a) comprehensive, given that it includes more than five times as many effect sizes as Dufner et al. (*k* = 54) and at least five times as many effect sizes as Blasco-Belled et al. (*k* ranges 3–52), (b) examines cross-cultural differences, and (c) tests self-esteem as a mechanism.

## Overview

We conducted an extensive and cross-cultural meta-analysis. We tested the associations of grandiose and vulnerable narcissism with wellbeing. We also tested whether these associations were explained by self-esteem and moderated by country-level individualism. We adopted an exploratory approach regarding all other moderators. In particular, we explored whether the relations of grandiose and vulnerable narcissism with wellbeing varied depending on (a) wellbeing forms (hedonic vs. eudaimonic), (b) the sample characteristics of age (given that narcissism wanes by age; Orth et al., 2024) as well as gender (given that narcissism is more prevalent in men than women; Grijalva, Newman, et al., 2015), and (c) the study characteristics of publication status (whether a study is published or unpublished), study design (whether the design is cross-sectional or longitudinal), publication year, and effect size type (whether it is zero-order or controlling for self-esteem).

## Method

### Transparency and Openness

We complied with Transparency and Openness Promotion Guidelines. We stored data, analysis codes, and research materials at OSF: https://osf.io/buaf7/?view_only=f7855069a9f7444f95090546f0bec433. The meta-analysis was not preregistered.

### Literature Search

We sought to achieve sufficient variability in country-level individualism. For this reason, we conducted literature searches in three English databases (Google Scholar, PsycINFO, Web of Science) and three Chinese databases (Chinese National Knowledge Infrastructure, Chongqing VIP information, Wanfang Data). We included literature from Chinese databases to account for the underrepresentation of samples from Western, Educated, Industrialized, Rich, and Democratic (WEIRD) countries in English databases. We searched for both published and unpublished studies (dissertations, master’s theses, conference presentations) to minimize the influence of publication bias. We carried out the initial search in the English databases in February 2020, and updated it in July 2022. We searched the Chinese databases in July 2022.

In our search, we used multiple keywords referring to narcissism and wellbeing in different combinations. Keywords referring to narcissism were: *narcissism, narcissistic personality disorder, NPD, dark triad, egotism*, and *cluster B personality.* Keywords referring to wellbeing were: *wellbeing* (*well being* and *well-being*), *life satisfaction, happiness, pleasure, contentment, joy, quality of life, positive affect/emotion/mood, hedonia, eudaimonia, positive life function, vitality, fulfillment, meaning of/in life*, and *purpose of/in life*. We translated these keywords to Chinese prior to searching the Chinese databases (see Supplemental Material Section A for the full search strings).

### Screening

We set four inclusion criteria. *First*, the studies should measure both narcissism and wellbeing, with no restriction on the operationalization of these constructs. *Second*, the studies should report at least one association (zero-order correlation) between narcissism and wellbeing. Given that we aimed to explore the role of self-esteem in the association between narcissism and wellbeing, we also included studies reporting associations between narcissism and wellbeing in which self-esteem was controlled for (i.e., partial correlations).^4^ *Third*, the studies should test more than one participant, implying that we excluded case studies. *Fourth*, for intervention studies to be incorporated, we ought to be able to extract baseline and control condition results.

**Figure 1.**
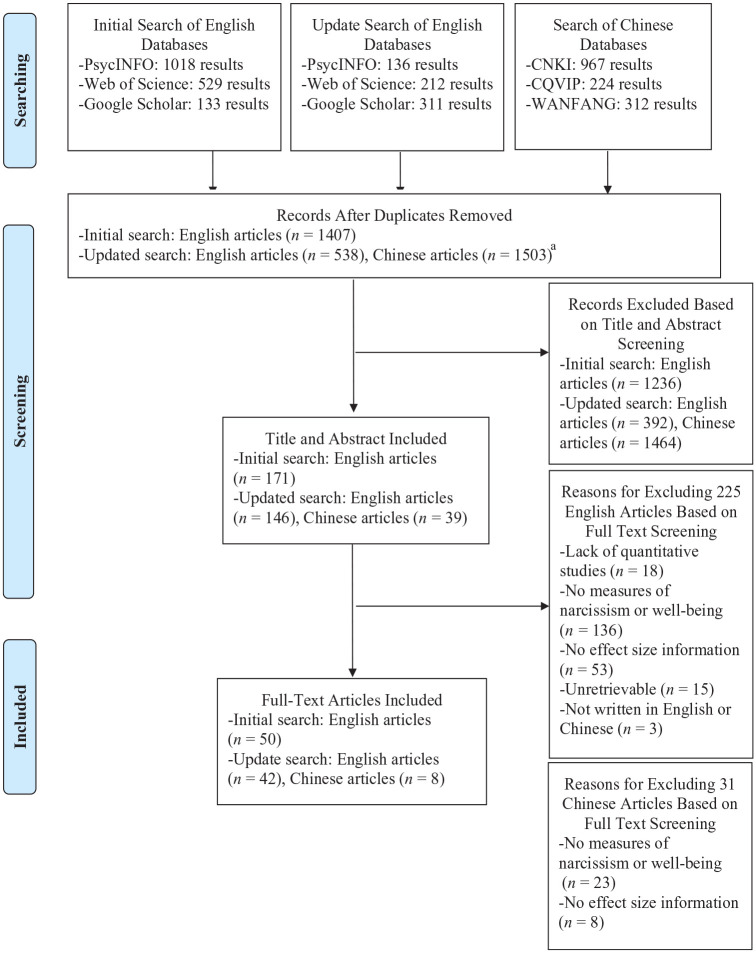
Flowchart of Literature Search and Screening. ^a^ We used Rayyan (Ouzzani et al., 2016) for deduplicating English articles. However, at the time of our screening, deduplicating Chinese articles via Rayyan proved ineffective due to difficulties in recognizing Chinese characters (i.e., they were displayed as gibberish). Consequently, we manually screened and reviewed each Chinese article.

Figure 1 presents a flowchart. We adopted a two-step screening procedure to determine the eligibility of the identified articles. The first step involved screening each study’s title and abstract. Two raters independently screened 10% of the identified articles. Interrater agreement was 94% in the initial search (February 2020) and 92% in the updated search (July 2022). The raters resolved disagreements through discussion. Then, each rater proceeded to screen the titles and abstracts of half of the remaining search results. The second step involved screening each study’s full text. Two raters independently screened the full text of 10% of the search results. Interrater agreement was 95% in the initial search (February 2020) and 90% in the updated search (July 2022). The raters sorted out disagreements via discussion. Subsequently, each rater proceeded to screen the full text of half of the remaining search results. For the Chinese studies, we carried out the same two-step screening procedure. Two raters independently screened 10% of the titles/abstracts and full texts for eligibility, reaching a good interrater agreement in both screening steps (94% and 100%, respectively). Again, the raters sorted out any disagreement through discussion.

This screening procedure yielded 100 articles that were eligible for inclusion (see Supplemental Material Section B for the references). Of them, 92 were written in English and 8 in Chinese (see Supplemental Material Section C for an overview of included studies).

### Coding of Studies

For each included study, we coded effect sizes, variables of main interest, as well as sample and study characteristics.

#### Effect Size

For the main analysis, we extracted zero-order correlation coefficients (*r*) that indicated the association between narcissism and wellbeing. If studies reported effect sizes for both the total sample and subgroups (e.g., women and men separately), we included effect sizes for subgroups. We did so for every subgroup (i.e., not only for those reflecting covariates or potential moderators).

For the self-esteem analysis, we additionally included partial correlations and regression coefficients (β) that were controlled for self-esteem to explore the role of self-esteem in the association between narcissism and wellbeing. We did not extract betas derived from hierarchical regression models and structural equational models, because these techniques adopt different approaches from the linear regression model for effect estimation. We did include betas derived from mediation and moderation models that were built with the macro Process (Hayes, 2013), as these are estimated using least-square regressions. We used the imputation formula *r* = β + .05λ (Peterson & Brown, 2005) to transform regression coefficients (β) to correlations (*r*). In this equation, λ equals 1 when β is nonnegative and 0 when β is negative.

#### Variables of Main Interest

##### Narcissism Forms

We coded narcissism as *grandiose* or *vulnerable* based on information that the authors provided in each primary study. When this information was missing, we examined the content of the measures used and made an informed and consensual decision about narcissism form. In very few instances, we were unable to classify measures as reflecting grandiose or vulnerable narcissism (e.g., Edelstein et al., 2012), and therefore we excluded the pertinent studies from further analyses (see Supplemental Material Section D for the categorization of narcissism measures).

##### Wellbeing Forms

We coded wellbeing as *hedonic, eudaimonic*, or *mixed*.^5^ We coded positive affect and life satisfaction as indicators of *hedonic wellbeing*. We coded meaning in life, purpose in life, spirituality, sacredness, religious wellbeing, fulfillment, flourishing, vitality, and basic psychological needs satisfaction as indicators of *eudaimonic wellbeing.* We coded *mixed wellbeing* when studies measured wellbeing with indicators of both *hedonic* and *eudaimonic wellbeing* (see Supplemental Material Section E for the categorization of wellbeing measures).

##### Individualism

We coded country-level individualism based on Hofstede et al.’s (2010) individualism index, with higher scores indicating greater individualism. Specifically, we coded country-level individualism based on a sample’s demographic information. If a sample was derived from several countries, we used the mean values of these countries’ levels of individualism. Whenever the country of a given study was not reported, we coded the country of the first author’s affiliation.

#### Sample and Study Characteristics

We coded the sample characteristics of mean age and gender (percentage of female participants). Also, we coded the study characteristics of publication status (published vs. unpublished), study design (cross-sectional vs. longitudinal), publication year, and effect size type (zero-order vs. controlling for self-esteem). See Supplemental Material Section G for the descriptive statistics and correlations among moderators.

#### Coding Reliability

Two raters independently coded 10% of the included studies. In the initial search, intraclass correlations for continuous variables ranged from .99 to 1, and Cohen’s κ for categorical variables ranged from .75 to 1. In the updated search, intraclass correlations ranged from .99 to 1, and Cohen’s κ for categorical variables ranged from .71 to 1. The raters settled all disagreements via discussion. Each rater proceeded to code half of the remaining studies.

### Data Analysis

Prior to data analyses, we converted all correlations *r* to Fisher’s *z* values. The Fisher’s *z* values can normalize the potentially skewed distribution of bivariate correlations before aggregation (Lipsey & Wilson, 2001). After conducting the analyses, we transformed the Fisher’s *z* values back into Pearson’s *r* for interpretability. We interpreted effect sizes of *r* ≥ .05 as very small,^6^ *r* ≥ .10 as small, *r* ≥ .20 as medium, *r* ≥ .30 as large, and *r* ≥ .40 as very large (Funder & Ozer, 2019). For the moderator analyses, we mean-centered continuous variables and recoded categories of discrete variables into dummy variables. We used two-tailed tests, with α = .05, unless specified otherwise.

#### Model Construction

We carried out a three-level meta-analysis (Van den Noortgate et al., 2013, 2014) following the relevant procedure outlined by Assink and Wibbelink (2016). This approach allowed us to include multiple effect sizes nested in individual studies while accounting for dependency in effect sizes by modeling the hierarchical structure of the data. Given that these two forms of narcissism were theoretically related to wellbeing in opposing directions, the potential moderating effect of the coded variables on the association between narcissism and wellbeing might be different for grandiose and vulnerable narcissism. Therefore, we conducted two identical sets of meta-analysis, one for each narcissism form.

For the main analyses, we first estimated an overall association between narcissism and wellbeing in an intercept-only model. We applied the robust variance estimation method as a safeguard against model misspecification (Assink & Wibbelink, 2024). Then, we carried out two separate one-sided log-likelihood ratio tests to determine whether the within-study variance (at Level 2 of the model) and the between-study variance (at Level 3 of the model) in effect sizes were significant. These tests were one-tailed, because variance components can only deviate from zero in a positive direction (Assink & Wibbelink, 2016). In case of significant heterogeneity, we conducted bivariate moderator analyses to test whether the strength of the association between narcissism and wellbeing varied across potential moderators. Finally, we used the full dataset (pooling the grandiose and vulnerable narcissism effect sizes together) to test the interaction between individualism and narcissism forms. We controlled for the study and sample characteristics that were identified as moderators in the bivariate moderator analyses.

For the self-esteem analysis, we first estimated the overall association between narcissism and wellbeing, and then tested the within-study and between-study variance. In case of significant heterogeneity, we conducted bivariate moderator analyses to examine whether the strength of the association between narcissism and wellbeing varied across effect size type (zero-order vs. controlling for self-esteem).

#### Risk of Bias Assessment

Publication bias can cause inflated effect size estimates (Borenstein et al., 2009). We attempted to minimize this bias by including both published and unpublished studies. Furthermore, we inspected a funnel plot, which plots effect sizes against their standard error. Publication bias would introduce asymmetry in the plot, because nonsignificant and negative effect sizes are less likely to be published (Borenstein et al., 2009). We quantify asymmetry in the funnel plot through Egger’s regression test (Egger et al., 1997), the trim-and-fill method (Duval & Tweedie, 2000), and the Precision-Effect Test and Precision-Effect Estimate with Standard Errors (PET-PEESE) technique (Stanley & Doucouliagos, 2014). The accuracy of these methods has not been extensively researched in the case of three-level meta-analyses with dependent effect sizes. Accordingly, we interpreted the resulting effect sizes not as corrected, but as indicators of a plausible range of effect sizes that are missing in our literature search (Carter et al., 2019; Coburn & Vevea, 2015; Terrin et al., 2003).

## Results

### Included Studies

In total, we identified 284 effect sizes obtained from 123 independent samples, with an aggregate sample size of 52,533 participants. Sample size ranged from *N* = 49 to *N* = 4,340 (*M* = 369.90, *SD* = 420.74, *Mdn* = 262). Sample mean age (reported for 77.82% of the samples) ranged from 11.50 to 57.70 years (*M* = 27.29, *SD* = 9.58, *Mdn* = 24.50). These samples spanned 28 countries/regions, representing a diverse distribution of country-level individualism (Table 1). A substantial number of effect sizes (*n* = 59, comprising 20.77% of total effect sizes) were derived from countries/regions scoring relatively low^7^ (<55) on Hofstede’s individualism index (e.g., China, Pakistan, Romania, Turkey, Serbia, Slovenia, South Korea, Vietnam), whereas the rest of the effect sizes were derived from countries/regions scoring relatively high (>55) on Hofstede’s individualism index (*n* = 220, comprising 77.46% of total effect sizes).

**Table 1.** Distribution of Effect Sizes by Countries/Regions and Corresponding Individualism Levels.

Countries	Number of effect sizes	Mean sample size	Hofstede individualism score	Range of publication years
Australia	5	355.8	90	2021
Botswana	1	627	-	2018
Brunei	3	277	-	2020
Canada	19	211.79	80	2016–2022
China	33	748.03	20	2008–2022
China Hongkong	2	179	25	2014
Germany	19	442.63	67	2016–2021
Global^a^	1	541	-	2018
Greece	2	361	35	2010
Hungary	1	4,340	80	2022
India	5	175.4	48	2018–2020
Iran	1	223	41	2014
Israel	1	108	54	2017
Italy	3	460	76	2019–2020
Netherlands	6	173.67	80	2008–2020
Norway	2	214	69	2019
Pakistan	1	233	14	2020
Poland	38	286.58	60	2009–2021
Romania	5	535	30	2020
Serbia	1	439	25	2021
Slovenia	1	495	27	2021
South Korea	3	579	18	2021
Switzerland/Germany^b^	10	246	68	2017
Turkey	3	526.33	37	2015–2021
United Kingdom	2	111	89	2017
United States	114	293.53	91	1998–2022
United States/Germany^b^	1	63	79	2007
Vietnam	1	420.00	20	2022

*Note.* ^a^ One study did not report specific country information. Most participants were located in North America (61%) or Europe (26%). We were unable to calculate the individualism score without the specific country information, and so we could not include this study in the individualism moderation analysis, although we included it in other analyses. ^b^ Two studies recruited sample from two countries. We averaged the Hofstede individualism scores of the two countries.

### Grandiose Narcissism and Wellbeing

We based the meta-analysis of (a) grandiose narcissism and wellbeing, and (b) moderators of the relation between grandiose narcissism and wellbeing, on 223 zero-order effect sizes that we extracted from 120 studies (*N* = 52,068^8^). The intercept-only model yielded a small-to-medium overall effect size, *r* = .19, 95% confidence interval, CI [.16, .21], *p* < .001, indicating that higher grandiose narcissism is related to greater wellbeing. We found a heterogeneous distribution of effect sizes, both within studies (i.e., variance at Level 2), χ^2^(1) = 173.77, *p* < .001, and between studies, χ^2^(1) = 19.56, *p* < .001. Therefore, we carried out moderation analyses (Table 2).

**Table 2.** Results of Bivariate Moderator Analyses in the Association Between Grandiose Narcissism and Wellbeing.

	*S*	*k*	β_0_ (95% CI)	ES*r*	β_1_ (95% CI)	*F*(df_1_, df_2_)
Hofstede individualism	117	218	0.19 (0.16, 0.21)***	.19	0.001 (0.0001, 0.002)**^a^	4.72 (1, 216)*
Wellbeing forms	120	223	-	-	-	1.22 (2, 220)
Eudaimonic (RC)	24	38	0.17 (0.12, 0.23)***	.17	-	-
Hedonic	102	175	0.19 (0.16, 0.21)***	.19	0.01 (−0.04, 0.07)	-
Mixed	8	10	0.26 (0.16, 0.36)***	.25	0.09 (−0.02, 0.20)	-
Age mean	93	175	0.18 (0.15, 0.21)***	.18	−0.001 (−0.004, 0.003)^a^	0.22 (1, 173)
Female participants (%)	104	196	0.19 (0.16, 0.22)***	.19	0.001 (−0.001, 0.002)^a^	0.86 (1, 194)
Publication status	120	223	-	-	-	1.32 (1, 221)
Unpublished (RC)	11	19	0.24 (0.15, 0.33)***	.24	-	-
Published	109	204	0.18 (0.16, 0.21)***	.18	−0.06 (−0.15, 0.04)	-
Study year	120	223	0.19 (0.16, 0.22)***	.19	−0.005 (−0.01, 0.0004)^a^	3.81 (1, 221)
Study design	120	223	-	-	-	0.22 (1, 221)
Cross-sectional study (RC)	115	204	0.19 (0.16, 0.22)***	.19	-	-
Longitudinal study	7	19	0.17 (0.07, 0.26)*	.17	−0.02 (−0.12, 0.08)	-

*Note. s* = number of independent studies; *k* = number of effect sizes; β_0_ = intercept/mean effect size, Fisher’s *z*; ES*r* = effect size, *r*; β_1_ = estimated regression coefficient; CI = confidence interval; *F*(df1, df2) = omnibus test; RC = reference category.

a Here, and throughout this article, we represent very small effects with three decimals.

* *p* < .05. **p < .01. ***p < .001.

#### Moderators of the Relation Between Grandiose Narcissism and Wellbeing

##### Individualism

Individualism moderated the association between grandiose narcissism and wellbeing, *F*(1, 216) = 4.72, *p* = .031. This association was stronger in samples from countries with higher (than lower) levels of individualism, β_1_ = 0.001, 95% CI = [0.0001, 0.002], *p* = .031. The association was of medium size in countries/regions high on individualism, *r* = .20, 95% CI = [.18, .23], *p* < .001, and small-to-medium size in countries/regions low on individualism, *r* = .13, 95% CI = [.07, .19], *p* < .001. (We presented the cut-off points in the Included Studies section.)

##### Wellbeing Forms

Wellbeing forms did not moderate the association between grandiose narcissism and wellbeing, *F*(2, 220) = 1.22, *p* = .296. This association was similar for hedonic wellbeing, *r* = .19, 95% CI = [.16, .21], *p* < .001, eudaimonic wellbeing, *r* = .17, 95% CI = [.12, .23], *p* < .001, and mixed wellbeing, *r* = .25, 95% CI = [.16, .35], *p* < .001. Pairwise comparisons suggested that grandiose narcissism was comparably associated with all wellbeing forms (see Supplemental Material Section H for bivariate moderator analyses with pairwise comparison).

##### Sample Characteristics

*Age* did not moderate the association between grandiose narcissism and wellbeing, *F*(1, 173) = 0.22, *p* = .640. This association did not vary across samples of different ages, β_1_ = −0.001, 95% CI = [−0.004, 0.003]. Likewise, *gender* did not moderate the association between grandiose narcissism and wellbeing, *F*(1, 194) = 0.86, *p* = .354. The association did not differ across samples with varying proportions of female participants, β_1_ = 0.001, 95% CI = [−0.001, 0.002].

##### Study Characteristics

*Publication status* did not moderate the association between grandiose narcissism and wellbeing, *F*(1, 221) = 1.32, *p* = .352. The association was similar for published studies, *r* = .18, 95% CI = [.16, .21], *p* < .001, and unpublished studies, *r* = .24, 95% CI = [.15, .33], *p* < .001. Likewise, *study design* did not moderate the association between grandiose narcissism and wellbeing, *F*(1, 221) = 0.22, *p* = .637. The association was similar for cross-sectional studies, *r* = .19, 95% CI = [.16, .22], *p* < .001, and longitudinal studies, *r* = .17, 95% CI = [.07, .25], *p* = .001. *Publication year* did not moderate the association between grandiose narcissism and wellbeing, *F*(1, 223) = 3.81, *p* = .052. This association did not vary across publication years, β_1_ = −0.005, 95% CI = [−0.01, 0.0004].

#### The Role of Self-Esteem in the Relation Between Grandiose Narcissism and Wellbeing

We based our self-esteem analysis on 242 effect sizes extracted from 120 studies (see Supplemental Material Section F for the categorization of self-esteem measures). Of these effect sizes, 223 were zero-order and 19 were partial (i.e., controlling for self-esteem). Of the 19 partial correlations, 18 effect sizes only controlled for self-esteem and 1 effect size controlled for self-esteem plus coping flexibility. We observed a heterogeneous distribution of effect sizes, both within studies (i.e., variance at Level 2), χ^2^(1) = 215.31, *p* < .001, and between studies, χ^2^(1) = 19.37, *p* < .001. The effect size type (i.e., zero-order vs. controlling for self-esteem) moderated the association between grandiose narcissism and wellbeing, *F*(1, 240) = 22.83, *p* < .001, such that this association was not significant when controlling for self-esteem, *r* = .005, 95% CI = [−.07, .08], *p* = .901, but was significant and small-to-medium in size when not controlling for self-esteem, *r* = .19, 95% CI = [.16, .22], *p* < .001.

#### Sensitivity Analyses

We searched for outliers using the “influence” command of the metafor package (Viechtbauer, 2010). We identified two large negative effect sizes (*r* = −.36, −.40) as potential outliers in the association between grandiose narcissism and wellbeing, based on significant DFFITS values (indicating a difference in the predicted average effect when these effect sizes were included versus excluded in model fitting; Viechtbauer & Cheung, 2010). The sample sizes associated with these effect sizes were 434 and 233. We carefully examined the effect size coding of these studies and found no indications of errors or implausible values. Therefore, we decided to retain all effect sizes in the meta-analytic dataset. This decision aligns with previous research (Orth et al., 2021) and methodological literature that discourages the routine exclusion of studies solely based on extremely large or small effect sizes (Viechtbauer & Cheung, 2010). Nevertheless, we conducted a sensitivity analysis to investigate the potential influence of outliers in our analyses. See Supplemental Material Section I for the sensitivity analyses winsorizing outliers. (This ancillary analysis replaces the outliers [*z* > 3.29] with effect sizes whose *z* score = 3.29.) Our main findings remained robust after adjusting the outliers.

#### Publication Bias

**Figure 2.**
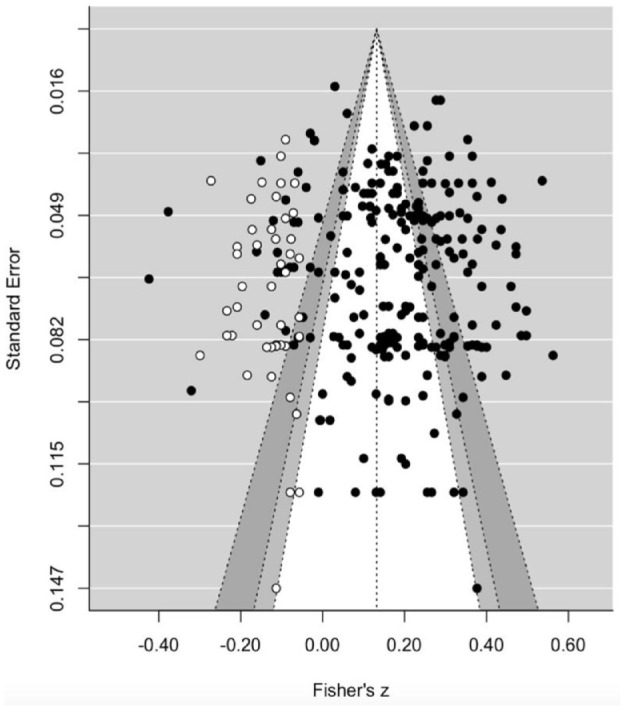
Funnel Plot for the Meta-Analysis on Grandiose Narcissism and Wellbeing. *Note.* Effect sizes (*x*-axis) are plotted against their standard errors (*y*-axis). The black dots denote observed effect sizes, whereas the white dots denote imputed effect sizes. The solid vertical line represents the overall mean effect. From inside to outside, the dashed lines limit the 90%, 95%, and 99% pseudo confidence interval regions.

The Egger’s regression test revealed that the funnel graph did not deviate significantly from a symmetrical shape, *z* = 1.55, *p* = .121, suggesting no significant publication bias. The trim-and-fill algorithm indicated that 46 effect sizes needed to be imputed to the left side of the plot to optimize symmetry (Figure 2). Accordingly, the adjusted overall effect size decreased to *r* = .13, 95% CI = [.11, .15], *p* < .001, which was slightly lower than the initially estimated overall correlation (Δ*r* = .06) between grandiose narcissism and wellbeing. As the effect size obtained from the PET model was significant, we proceeded with the PEESE model (Stanley & Doucouliagos, 2014). The PEESE-corrected effect size, β_0_ = 0.15, 95% CI [0.12, 0.18], *p* < .001, was slightly lower (Δ*r* = .04) than the initial estimate overall correlation between grandiose narcissism and wellbeing. The significant slope, β_1_ = 8.42, *p* = .046, indicated the presence of publication bias, but the very low *R*^2^ value of .01 suggests that the magnitude of this bias is minimal. Taken together, these findings did not produce evidence of substantial publication bias.

#### Summary

Elevated grandiose narcissism was associated with enhanced wellbeing. This association was moderated by individualism, such that it was more pronounced in countries higher on individualism. Moreover, this association became nonsignificant after controlling for self-esteem.

### Vulnerable Narcissism and Wellbeing

We based the meta-analysis of vulnerable narcissism and wellbeing on 61 effect sizes extracted from 37 studies (*N* = 10,592). The intercept-only model yielded a medium-to-large overall effect size, *r* = −.25, 95% CI [−.29, −.21], *p* < .001, indicating that higher vulnerable narcissism is related to decreased wellbeing. We observed a heterogeneous distribution of effect sizes, both within studies (i.e., variance at Level 2), χ^2^(1) = 12.38, *p* < .001, and between studies, χ^2^(1) = 11.68, *p* < .001. Therefore, we proceeded with moderation analyses (Table 3).

**Table 3.** Results of Bivariate Moderator Analyses in the Association Between Vulnerable Narcissism and Wellbeing.

	*s*	*k*	β_0_ (95% CI)	ES*r*	β_1_ (95% CI)	*F*(df_1_, df_2_)
Hofstede individualism	37	61	−0.26 (−0.30, −0.21)***	−.25	−0.001 (−0.002, 0.001)^a^	0.70 (1, 59)
Wellbeing forms	37	61	-	-	-	1.06 (2, 58)
Eudaimonic (RC)	10	15	−0.31 (−0.39, −0.22)***	−.30	-	-
Hedonic	26	42	−0.24 (−0.29, −0.19)***	−.24	0.07 (−0.02, 0.16)	-
Mixed	4	4	−0.27 (−0.41, −0.13)***	−.26	0.04 (−0.13, 0.20)	-
Age mean	29	46	−0.27 (−0.30, −0.23)***	−.26	−0.007 (−0.01, −0.003)**	10.20 (1, 44)**
Female participants (%)	33	54	−0.28 (−0.32, −0.23)***	−.27	0.0001 (−0.003, 0.003)^a^	0.002 (1, 52)^a^
Publication status	37	61	-		-	0.05 (1, 59)
Unpublished (RC)	4	6	−0.27 (−0.41, −0.13)***	−.26	-	-
Published	33	55	−0.25 (−0.30, −0.21)***	−.25	0.02 (−0.13, 0.16)	-
Publication year	37	61	−0.26 (−0.30, −0.22)***	−.25	0.005 (−0.003, 0.01)^a^	1.50 (1, 59)
Study design	37	61	-		-	2.88 (1, 59)
Cross-sectional study (RC)	35	53	−0.25 (−0.29, −0.20)***	−.25	-	-
Longitudinal study	2	8	−0.39 (−0.56, −0.23)***	−.37	−0.14 (−0.32, 0.03)	-

*Note. s* = number of independent studies; *k* = number of effect sizes; β_0_ = intercept/mean effect size, Fisher’s *z*; ES*r* = effect size, *r*; β_1_ = estimated regression coefficient; CI = confidence interval; *F* (df1, df2) = omnibus test; RC = reference category.

a Estimates represent very small effects.

* *p* < .05. **p < .01. ***p < .001.

#### Moderators of the Relation Between Vulnerable Narcissism and Wellbeing

##### Individualism

Individualism did not moderate the association between vulnerable narcissism and wellbeing, *F*(1, 59) = 0.70, *p* = .405, indicating that this association did not vary across countries that differed on individualism, β_1_ = −0.001, 95% CI = [−0.002, 0.001].

##### Wellbeing Forms

Wellbeing forms did not moderate the vulnerable narcissism–wellbeing association either, *F*(2, 58) = 1.16, *p* = .321. This association was similar for hedonic wellbeing, *r* = −.24, 95% CI = [−.28, −.19], *p* < .001, eudaimonic wellbeing, *r* = −.30, 95% CI = [−.37, −.22], *p* < .001, and mixed wellbeing, *r* = −.26, 95% CI = [−.39, −.13], *p* = .001. Pairwise comparisons indicated that vulnerable narcissism was comparably associated with all wellbeing forms (see Supplemental Material Section H).

##### Sample Characteristics

*Age* moderated the negative association between vulnerable narcissism and wellbeing, *F*(1, 44) = 10.20, *p* = .003. This negative association was stronger in older (than younger) samples, β_1_ = −0.007, 95% CI = [−0.01, −0.003], *p* = .003. The association was large in a sample above the mean age, *r* = −.30, 95% CI = [−.39, −.21], *p* < .001, and medium-to-large in a sample below the mean age, *r* = −.23, 95% CI = [−.28, −.19], *p* < .001. Finally, *gender* did not moderate the association between vulnerable narcissism and wellbeing, *F*(1, 52) = 0.002, *p* = .966. The association did not differ across samples with varying proportions of female participants, β_1_ = 0.0001, 95% CI = [−0.003, 0.003].

##### Study Characteristics

*Publication status* did not moderate the association between vulnerable narcissism and wellbeing, *F*(1, 59) = 0.05, *p* = .820. The association was similar for published studies, *r* = −.25, 95% CI = [−.29, −.21], *p* < .001, and unpublished studies, *r* = −.26, 95% CI [−.39, −.13], *p* < .001. Likewise, *study design* did not moderate the association between vulnerable narcissism and wellbeing, *F*(1, 59) = 2.88, *p* = .095. The association was similar for cross-sectional studies, *r* = −.25, 95% CI = [−.28, −.20], *p* < .001, and longitudinal studies, *r* = −.37, 95% CI = [−.51, −.23], *p* < .001. *Publication year* did not moderate the association between vulnerable narcissism and wellbeing, *F*(1, 59) = 1.50, *p* = .226. The association did not vary across publication years, β_1_ = 0.005, 95% CI = [−0.003, 0.01].

#### The Role of Self-Esteem in the Relation Between Vulnerable Narcissism and Wellbeing

We based our self-esteem meta-analysis on 67 effect sizes extracted from 37 studies. Of those effect sizes, 61 were zero-order and 6 were partial (i.e., controlling for self-esteem). Of the six partial correlations, five effect sizes only controlled for self-esteem and one effect size controlled for self-esteem plus coping flexibility. We observed a heterogeneous distribution of effect sizes, both within studies (i.e., variance at Level 2), χ^2^(1) = 40.96, *p* < .001, and between studies, χ^2^(1) = 5.59, *p* = .009. Type of effect size (i.e., zero-order vs. controlling for self-esteem) moderated the association between vulnerable narcissism and wellbeing, *F*(1, 65) = 15.52, *p* < .001, such that this association was not significant when controlling for self-esteem, *r* = −.05, 95% CI = [−.16, .06], *p* = .363, but it was significant and medium-to-large in size when not controlling for self-esteem, *r* = −.21, 95% CI = [−.30, −.10], *p* < .001.

#### Sensitivity Analyses

We searched for outliers using the “influence” command of the metafor package (Viechtbauer, 2010). We did not identify any potential outliers in the association between vulnerable narcissism and wellbeing.

#### Publication Bias

**Figure 3.**
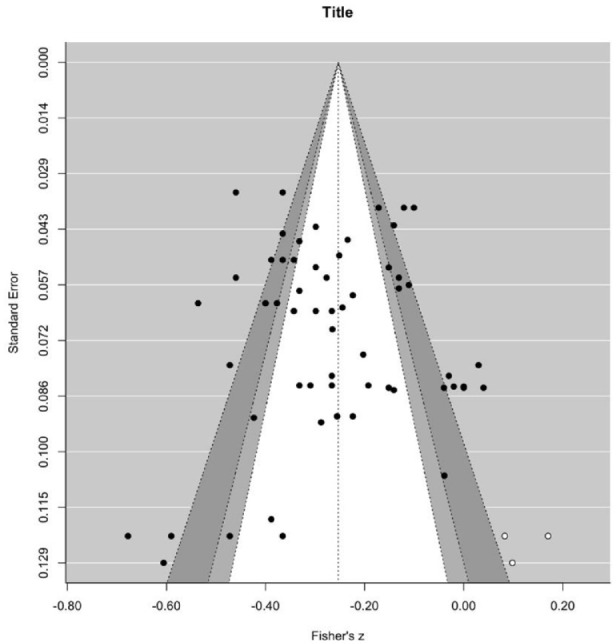
Funnel Plot for the Meta-Analysis on Vulnerable Narcissism and Wellbeing. *Note.* Effect sizes (*x*-axis) are plotted against their standard errors (*y*-axis). The black dots denote observed effect sizes, whereas the white dots denote imputed effect sizes. The solid vertical line represents the overall mean effect. From inside to outside, the dashed lines limit the 90%, 95%, and 99% pseudo confidence interval regions.

The Egger’s regression test revealed no significant publication bias, *z* = −0.81, *p* = .419. The trim-and-fill algorithm indicated that three effect sizes needed to be imputed to the right side of the plot to restore symmetry (Figure 3). The adjusted overall effect size, *r* = −.25, 95% CI = [−.29, −.21], *p* < .001, is almost identical to the initially estimated overall correlation between vulnerable narcissism and wellbeing. As the effect size obtained from the PET model was significant, we proceeded with the PEESE model. The PEESE-corrected effect size, β_0_ = −.26, 95% CI [−.33, .21], *p* < .001, was slightly larger (Δ*r* = .01) than the initial estimate overall correlation between vulnerable narcissism and wellbeing. The nonsignificant slope suggested no evidence of publication bias, β_1_ = −0.94, *p* = .884. Taken together, these analyses found little evidence for publication bias.

#### Summary

Elevated vulnerable narcissism was associated with lower wellbeing. This association was not significantly moderated by individualism but was moderated by age such that it was more negative among older (than younger) adults.

### Interaction Between Individualism and Narcissism Form

We based the analysis of the interaction between individualism and narcissism form on the full dataset. The analysis consisted of 284 effect sizes that were obtained from 123 independent samples, with an aggregate *N* = 52,533. Separate meta-analyses for each narcissism form indicated that *age* was a significant moderator of the relation between vulnerable narcissism and wellbeing. We thus controlled for *age* in this final model to arrive at an accurate estimate of the interaction between individualism and narcissism form. This interaction was significant, β_1_ = 0.003, 95% CI [0.001, 0.004], *p* = .010, indicating that the discrepancy between the two narcissism forms’ associations with wellbeing enlarges as individualism increases. This interaction was robust to the winsorization of outliers (see Supplemental Material Section I).

## Discussion

What does it feel like to be a narcissist? The association between narcissism and wellbeing has been the subject of theoretical and empirical scrutiny for decades, with some perspectives advocating that it is positive, and others that it is negative. We sought to address this conundrum by conducting a comprehensive meta-analysis of the literature separating grandiose from vulnerable narcissism, and investigating the extent to which the wellbeing benefits or liabilities of narcissism depend on person–culture fit. We hypothesized and found that grandiose narcissism is positively associated with both forms of wellbeing, a phenomenon accounted for by high self-esteem. As hypothesized, country-level individualism emerged as a moderator, with this association being stronger in countries high on individualism. Furthermore, we hypothesized and found that vulnerable narcissism is negatively associated with both wellbeing forms, a phenomenon accounted for by low self-esteem. Contrary to our hypothesis, country-level individualism did not moderate this association. Together, these findings underline the importance of separating grandiose from vulnerable narcissism in investigations of wellbeing, and of examining the fit between a person’s grandiose narcissism levels and their cultural context.

### Theoretical Implications

Grandiose narcissism was associated with greater wellbeing, especially in countries higher on individualism. This finding is consistent with person–environment fit (Van Vianen, 2018) and culture–person fit (Gebauer et al., 2013) perspectives, suggesting that grandiose narcissism is conducive to wellbeing to the extent that it matches the type of traits that are valued in their cultural context. In individualistic cultures, grandiose narcissists may feel that their core tendencies of extraversion, approach orientation, and self-promotion are valued. As grandiose narcissists tend to be attracted to other grandiose narcissists (Grosz et al., 2015), it is also possible they are happier in individualist cultures simply because these cultures have more people like them (Cai et al., 2012; Li & Benson, 2022). By contrast, vulnerable narcissism was associated with poorer wellbeing, independent of a country’s level of individualism. One explanation for this finding is that vulnerable narcissism, unlike grandiose narcissism, maps onto neuroticism. Some researchers have even argued that vulnerable narcissism is mostly a disorder of neuroticism (Miller et al., 2018). Neuroticism is neither valued nor devalued more in cultures high (vs. low) on individualism. For this reason, the effects of vulnerable narcissism on wellbeing may not depend on a country’s individualism levels, whereas those of grandiose narcissism do.

Our findings were robust to a wide range of moderators. In particular, gender, publication status, and study design were not significant moderators. Furthermore, age did not moderate the relation between grandiose narcissism and wellbeing, but it did moderate the relation between vulnerable narcissism and wellbeing, with the latter relation being weaker in older (vs. younger) samples. That is, aging vulnerable narcissists were less susceptible to suffer from poor wellbeing. Also, publication year did not moderate the relation between grandiose narcissism and wellbeing or vulnerable narcissism and wellbeing. Finally, publication bias did not appear to influence the results.

The findings highlight a discrepancy between the interpersonal and intrapersonal worlds of grandiose narcissists. On the one hand, grandiose narcissists can be a source of trouble for others. That is, they create problems in their interpersonal relationships: Albeit appealing and exciting especially in the initial stages of the relationship (Foster & Twenge, 2011) and often seen as leaders (Brummelman et al., 2021; Grijalva, Harms, et al., 2015), they have an avoidant attachment style, are callous, are often unfaithful in their romantic relationships, and can be aggressive or violent (Campbell & Foster, 2002; Kjærvik & Bushman, 2021). Also, narcissists create problems in their organizations: They are status oriented and alienating (Grapsas et al., 2020; Roberts et al., 2018), and, as leaders, albeit intrepid and occasionally innovative, can be bullying, impulsive, prone to fraud, and financially reckless (Cragun et al., 2020; Sedikides & Campbell, 2017). On the other hand, narcissists enjoy good wellbeing. How can this discrepancy be explained?

It is possible that, due to a combination of self-absorption, self-centeredness, and high self-esteem, grandiose narcissists coax themselves into thinking that others perceive them as favorably as they perceive themselves, contributing to a wellbeing nirvana. Yet, even grandiose narcissists seem aware that their reputations sour over time (Carlson et al., 2011). It is also possible that, due to their callousness, contentiousness, manipulativeness, and lack of empathy, they are indifferent or blame others for their problems, absolving themselves of responsibility and hence maintaining good wellbeing (McAllister et al., 2002; Stucke, 2003). Regardless, these explanations raise the possibility that the wellbeing of grandiose and vulnerable narcissists is an antecedent or consequence of their social behavior, a possibility worth testing.

Vulnerable narcissists suffered poor wellbeing, as per their low self-esteem, whereas grandiose narcissists enjoyed good wellbeing, as per their high self-esteem. What are the sources of grandiose narcissists’ high self-esteem? One source is their belief in their intellectual ability. Indeed, narcissistic self-esteem drops in tandem with lowered perceptions of their intelligence (Zajenkowski et al., 2022). Other sources of narcissistic self-esteem are status (Grapsas et al., 2020), perceived physical attractiveness (Vazire et al., 2008), and power (Zeigler-Hill & Beigi Dehagh, 2023). That said, our work focused specifically on self-esteem as a mechanism, and does not rule out alternative mechanisms (e.g., extraversion and neuroticism, which are both associated with grandiose narcissism, vulnerable narcissism, and wellbeing; Hyatt et al., 2018; Zeigler-Hill & Vrabel, 2022). Future research could compare self-esteem to other mechanisms explaining how grandiose and vulnerable narcissism enjoy different levels of wellbeing.

Our findings raise a practical implication. Psychologists (Diener et al., 2009), behavioral scientists (Graham et al., 2018), and utilitarian philosophers (Parfit, 2017) have called for strategies to maximize wellbeing at the societal level. Should such a socio-cultural agenda involve raising narcissism at the individual level as well? We would not advocate this option. First, our findings are correlational, and so the direction of causation is uncertain. Second, apart from associations with wellbeing, narcissism can be problematic for interpersonal relationships (Czarna et al., 2022) and, when in leadership positions, for organizations or even countries (Harden, 2021; Sedikides & Campbell, 2017). Third, our findings reveal that the benefits of grandiose narcissism to wellbeing are explained by self-esteem. Thus, a safer practice would be to raise self-esteem without breeding narcissism (Brummelman & Sedikides, 2020).

### Strengths, Limitations, and Future Directions

Our work has several limitations. First, we excluded negative affect as to not confound it with subclinical psychopathology. A stream of evidence indicates that long-term (i.e., trait) positive affect is separate, but not necessarily independent, from long-term negative affect (Payne & Schimmack, 2024). A future meta-analysis may examine the connection between narcissism and ill-being, focusing on negative affect as well as indices of subclinical psychopathology.

Second, we did not control for social desirability. Many primary grandiose narcissism studies assessed this construct with the forced-choice response version of the Narcissistic Personality Inventory (Raskin & Terry, 1988), a format that constraints socially desirable responding (Sedikides et al., 2004; Watson et al., 1984). Yet, socially desirable responding has occasionally been reported using a forced-choice response format (Kowalski et al., 2018) and different narcissism scales (Barry et al., 2017). Two studies implementing a bogus pipeline paradigm (Jones & Sigall, 1971), an experimental technique to reduce socially desirable responding, are also informative. In one, grandiose narcissists reported lower self-esteem in the bogus pipeline (vs. control) condition (Myers & Zeigler-Hill, 2012). In the other, vulnerable narcissists were more likely to admit being sad in the bogus pipeline (vs. control) condition, a pattern that did not emerge among grandiose narcissists (Brunell & Buelow, 2019). In the current meta-analysis, very few primary studies assessed social desirability (mostly in regard to grandiose narcissism), which is why we opted against inclusion of this construct. Relatedly, another meta-analysis reported null findings regarding the relation between self-esteem and socially desirable responding (Huang, 2013). Regardless, we recommend that researchers systematically use a social desirability scale when addressing the link between narcissism and wellbeing.

Third, self-esteem was controlled for in a limited number of cases. Specifically, we analyzed only 19 partial correlations regarding the association between grandiose narcissism and wellbeing, and only six partial correlations regarding the association between vulnerable narcissism and wellbeing, controlling for self-esteem. We recommend that researchers systematically measure self-esteem when studying the association between narcissism and wellbeing. Relatedly, for the self-esteem analysis, we focused on synthesizing partial correlations to address the controlled relations between narcissism and well being. An alternative approach is a meta-analytic structural equation modeling (MASEM), which combines correlation matrices to fit path or structural equation models on the pooled data (Jak et al., 2021). MASEM could allow for a more comprehensively evaluation of direct and indirect relations between narcissism and wellbeing, mediated by self-esteem. The examination of partial correlations, however, has an intuitive advantage. In addition, MASEM is unlikely to provide a substantial improvement in the accuracy of our estimates. Regardless, future meta-analyses might consider adopting MASEM for testing hypotheses regarding meta-analytic mediation or partial effects.

Fourth, when analyzing country-level variables, a researcher would do well to consider spatial nonindependence (i.e., proximal countries being more similar than distant countries), because failing to do so can lead to biased estimates (Ebert et al., 2023). Spatial regression, which can be implemented in meta-analyses, is an answer (Ward & Gleditsch, 2008). This issue, though, is less likely to have impacted our study, given the global distribution of our samples.

Finally, our meta-analysis was limited by the available measures of eudaimonic wellbeing. Researchers have called for an expansion of the definition of eudaimonic wellbeing to incorporate character strengths (Seligman, 2002) and psychological richness (Oishi & Westgate, 2022). We hope that the future meta-analyses of this literature can rely on eudaimonic wellbeing measures that align with these recommendations as well as be able to take into account subdivisions of grandiose narcissism (e.g., admirative vs. rivalrous) and varying research designs (e.g., experimental, momentary ecological assessment).

More broadly, our work identifies new research directions. One such direction involves the examination of narcissism as a state rather than a trait. To date, grandiose and vulnerable narcissism have been studied primarily as traits. Yet, individuals can experience grandiose and vulnerable narcissism as states (Giacomin & Jordan, 2016). Whereas individuals who are dispositionally grandiose express both grandiosity and vulnerability, those who are dispositionally vulnerable have high levels of vulnerability and low levels of grandiosity (Edershile & Wright, 2021). A research stream may explore the consequences of fluctuations in grandiose and vulnerable states for wellbeing.

### Constraints on Generality

We conducted a comprehensive meta-analysis on narcissism and wellbeing, accounting for the underrepresentation of samples from non-WEIRD countries by searching for literature in both English and Chinese databases. Our samples derived from 28 countries/regions. Yet, we identify three constraints on generality. First, most samples were from countries relatively high on individualism (e.g., Australia, the Netherlands, United Kingdom, United States). About one-fifth of samples were from countries relatively low on individualism (e.g., China, Pakistan, Romania, Turkey, Serbia, Slovenia, South Korea, Vietnam). Second, only one sample was from Africa (i.e., Botswana). Also, we did not take into account diversity within non-WEIRD cultures (Krys et al., 2025); for example, the meta-analysis includes an overrepresentation of Chinese culture. Third, many samples had a low mean age, partly because they were drawn from student populations. Thus, our results may not readily be generalized to countries at the low end of the individualism spectrum, African countries, and older populations.

### In Closing

Whether narcissism contributes to wellbeing has been a controversial issue. We meta-analytically found that grandiose narcissism conduces to increased wellbeing (hedonic and eudaimonic), as per their high self-esteem, whereas vulnerable narcissism conduces to decreased wellbeing, as per their low self-esteem. Furthermore, the benefits of grandiose narcissism are most pronounced in countries high on individualism. These findings reconcile divergent theoretical perspectives, demonstrate the importance of person–culture fit in studying wellbeing, and reinforce the relevance of narcissism in predicting subjective experience.

## Supplemental Material

sj-docx-1-psp-10.1177_01461672241307531 – Supplemental material for Narcissism and Wellbeing: A Cross-Cultural Meta-Analysis Supplemental material, sj-docx-1-psp-10.1177_01461672241307531 for Narcissism and Wellbeing: A Cross-Cultural Meta-Analysis by Constantine Sedikides, Yixin Tang, Yan Liu, Eva de Boer, Mark Assink, Sander Thomaes and Eddie Brummelman in Personality and Social Psychology Bulletin

## Notes

1. We focused on the presence of well being rather than the absence of ill-being. There is considerable variability in the way hedonic well being is conceptualized or operationalized (Busseri & Sadava, 2011; Huta, 2017; Zhou et al., 2023), with some definitions incorporating negative affect (Busseri, 2018; Diener et al., 1999). Furthermore, we refrained from including negative affect for a pragmatic reason. We were concerned with confounding negative affect with subclinical psychopathology (e.g., depression, anxiety), given that most of the relevant primary articles assessed negative affect as part of subclinical psychopathology.

2. Grandiose narcissism has also been subdivided into agentic (reflecting self-enhancement on the agentic domain such as intelligence, creativity, or vision) and communal (reflecting self-enhancement on the communal domain such as warmth, prosociality, or morality; Gebauer et al., 2012). We were not concerned with grandiose communal narcissism, as we were unable to locate studies examining the relation between this form of narcissism and wellbeing.

3. Blasco-Belled et al. (2024) used the terms “subjective well being” for hedonic wellbeing, and “psychological well being” for eudaimonic wellbeing.

4. We only included zero-order correlations in the main analysis. We did not include betas, because they usually present partial correlations. For the self-esteem analysis, we did include partial correlations and betas.

5. We included mixed well being, because it was occasionally unclear which form of well being primary articles assessed or whether they adequately distinguished between the two forms.

6. As Funder and Ozer (2019) put it, “. . . an effect size of .05 is considered *very small* for the explanation of single events but potentially consequential in the not-very-long-run.”

7. We determined the Hofstede’s individualism index cut-off points (>55 and <55) after Tang et al. (2023).

8. For correlations among moderators and descriptive statistics, see Supplemental Material, Section G (Table 1S).
