# Supplementary material for: Narcissism and Wellbeing: A Cross-Cultural Meta-Analysis
Source: Pers Soc Psychol Bull. 2024 Dec 31;52(5):1222–38. doi: 10.1177/01461672241307531 (PMC13022027; doi:10.1177/01461672241307531)
Supplement: sj-docx-1-psp-10.1177_01461672241307531 – Supplemental material for Narcissism and Wellbeing: A Cross-Cultural Meta-Analysis [file sj-docx-1-psp-10.1177_01461672241307531.docx]

**SUPPLEMENTAL MATERIAL**

**Narcissism and Wellbeing: A Cross-Cultural Meta-Analysis**

[A. FULL SEARCH STRING 2](#_Toc182318637)

[B. REFERENCE LIST OF INCLUDED STUDIES 4](#_Toc182318638)

[C. OVERVIEW OF INCLUDED STUDIES 17](#_Toc182318639)

[D. CATEGORIZATION OF NARCISSISM MEASURES 29](#_Toc182318640)

[E. CATEGORIZATION OF WELLBEING MEASURES 40](#_Toc182318641)

[F. CATEGORIZATION OF SELF-ESTEEM MEASURES 51](#_Toc182318642)

[G. CORRELATIONS AMONG MODERATORS AND DESCRIPTIVE STATISTICS 52](#_Toc182318643)

[H. BIVARITE MODERATORS ANALYSES 53](#_Toc182318644)

[I. SENSITIVITY ANALYSIS WINSORIZING OUTLIERS 54](#_Toc182318645)

1. **FULL SEARCH STRING**
2. **PsycINFO**

#1 narcissism

Narcissism/ OR Narcissistic Personality Disorder/ OR (narcis* OR NPD OR dark triad OR cluster b personality disorder* OR egotism).ti,ab,id,tm.

#2 well-being

Well being/ OR life satisfaction/ OR happiness/ OR pleasure/ OR contentment/ OR "Quality of Life"/ OR (wellbeing OR well-being OR positive mood OR positive affect OR happiness OR happy OR pleasur* OR contentment* OR joy OR hedoni* OR eudaimoni* OR positive emotion* OR (quality ADJ2 life) OR QoL OR positive life functioning OR vitality OR fulfillment OR meaning in life OR meaning of life OR purpose in life OR purpose of life).ti,ab,id,tm.

1AND 2

1. **Web of Science**

#1 narcissism

TS=("narcis*" OR "NPD" OR "dark triad" OR "cluster b personality disorder*" OR "egotism")

#2 well-being

TS=("wellbeing" OR "well-being" OR "positive mood" OR "positive affect" OR "happiness" OR "happy" OR "pleasur*" OR "contentment*" OR "joy" OR "hedoni*" OR "eudaimoni*" OR "positive emotion*" OR ("quality" NEAR/1 "life") OR "QoL" OR "positive life functioning" OR "vitality" OR "fulfillment" OR "meaning in life" OR "meaning of life" OR "purpose in life" OR "purpose of life")

1 AND 2

1. **Google Scholar**

narcissism|egotism wellbeing|well-being|"positive affect"| happiness| happy|pleasure|contentment|joy|hedonia|eudaimonia|"positive emotion"|"quality of life"|"QoL"|"positive life functioning"|vitality| fulfillment|"meaning in life"|"meaning of life"|"purpose in life"|"purpose of life"

*Note*: The Chinese search strings are available upon request.

1. **REFERENCE LIST OF INCLUDED STUDIES**

Adeeb, M. (2020). Narcissism and life satisfaction in college students: Mediating role of social media addiction. J*ournal of Pakistan Psychiatric Society, 17*(3), 10-13.

Aghababaei, N., & Błachnio, A. (2015). Well-being and the Dark Triad. *Personality and Individual Differences*, *86*, 365–368. https://doi.org/10.1016/j.paid.2015.06.043

Aghababaei, N., Mohammadtabar, S., & Saffarinia, M. (2014). Dirty Dozen vs. the H factor: Comparison of the Dark Triad and Honesty–Humility in prosociality, religiosity, and happiness. *Personality and Individual Differences*, *67*, 6–10. https://doi.org/10.1016/j.paid.2014.03.026

Akinci, İ. (2015). *The relationship between the types of narcissism and psychological well-being: the roles of emotions and difficulties in emotion regulation*. [Master’s Thesis, Middle East Technical University]. Open METU. http://etd.lib.metu.edu.tr/upload/12619052/index.pRdf

Aminnuddin, N. A. (2020). Personality and Islamic religiosity: Preliminary survey data of Bruneian Malay Muslim university students and their psychological well-being, unethical behavior, and dark triad traits. *Data in Brief*, *30*, Article 105486. https://doi.org/10.1016/j.dib.2020.105486

Anderson, K., & Costello, P. (2009). *Relationships between prosocial behavior, spirituality, narcissism, and satisfaction with life.* [Bachelor’s Thesis, Gustavus Adolphus College]. Gustavus Adolphus College Repository. https://gustavus.edu/psychology/documents/KellyAndersonSpr09.pdf.

Atroszko, P. A., Balcerowska, J. M., Bereznowski, P., Biernatowska, A., Pallesen, S., & Schou Andreassen, C. (2018). Facebook addiction among Polish undergraduate students: Validity of measurement and relationship with personality and well-being. *Computers in Human Behavior*, *85*, 329–338. https://doi.org/10.1016/j.chb.2018.04.001

Ben Shlomo, S., & Taubman - Ben-Ari, O. (2017). What factors may assist social workers to promote life satisfaction and personal growth among first-time grandfathers? *Child &Family Social Work*, *22*(1), 482–491. https://doi.org/10.1111/cfs.12267

Błachnio, A., & Przepiórka, A. (2018). Facebook intrusion, fear of missing out, narcissism, and life satisfaction: A cross-sectional study. *Psychiatry Research*, *259*, 514–519. https://doi.org/10.1016/j.psychres.2017.11.012

Bogart, L. M., Benotsch, E. G., & Pavlovic, J. D. P. (2004). Feeling superior but threatened: The relation of narcissism to social comparison. *Basic and Applied Social Psychology*, *26*(1), 35–44. https://doi.org/10.1207/s15324834basp2601_4

Bourbonnais, K., & Durand, G. (2018). The incremental validity of the Triarchic model of psychopathy in replicating “The dark side of love and life satisfaction: Associations with intimate relationships, psychopathy and Machiavellianism”. *The Quantitative Methods for Psychology*, *14*(3), 12–17. https://doi.org/10.20982/tqmp.14.3.r001

Brailovskaia, J., & Margraf, J. (2016). Comparing Facebook users and Facebook non-users: Relationship between personality traits and mental health variables – An exploratory study. *PLoS ONE*, *11*(12), Article e0166999. https://doi.org/10.1371/journal.pone.0166999

Brailovskaia, J., & Margraf, J. (2019). I present myself and have a lot of Facebook-friends – Am I a happy narcissist!? *Personality and Individual Differences*, *148*, 11–16. https://doi.org/10.1016/j.paid.2019.05.022

Brunell, A. B., & Buelow, M. T. (2019). Using the bogus pipeline to investigate trait narcissism and well-being. *Personality and Individual Differences*, *151*, Article 109509. https://doi.org/10.1016/j.paid.2019.109509

Cai, P., Zuo, S., Wang, F., Huang Y., & Xu. Y. (2017). The effect of the dark triad personality on teenager's orientations to happiness and subjective well-being, *Chinese Journal of Special Education, 12*, 60-67.

Çevik, O., Koçak, O., Younis, M., & Çevik, E. (2021). The mediating role of gaming disorder in the effect of narcissism on happiness in children. *International Journal of Environmental Research and Public Health*, *18*(13), Article 7137. https://doi.org/10.3390/ijerph18137137

Chamberlain, J. M., & Haaga, D. A. F. (2001). Unconditional self-acceptance and psychological health. *Journal of Rational-Emotive and Cognitive-Behavior Therapy*, *19*(3), 163–176. https://doi.org/10.1023/a:1011189416600

Charzyńska, E., Sussman, S., & Atroszko, P. A. (2021). Profiles of potential behavioral addictions’ severity and their associations with gender, personality, and well-being: A person-centered approach. *Addictive Behaviors*, *119*, Article 106941. https://doi.org/10.1016/j.addbeh.2021.106941

Clark, M. A., Lelchook, A. M., & Taylor, M. L. (2010). Beyond the Big Five: How narcissism, perfectionism, and dispositional affect relate to workaholism. *Personality and Individual Differences*, *48*(7), 786–791. https://doi.org/10.1016/j.paid.2010.01.013

Ding, Q., Zhang, Y., Wei, H., Niu, G., & Zhou, Z. (2016). The relationship among narcissism, selfie posting behavior and positive affect in college students. *Chinese Journal of Clinical Psychology, 24*(3), 519–522. https://doi.org/10.16128/j.cnki.1005-3611.2016.03.030

Doehring, C., Clarke, A., Pargament, K. I., Hayes, A., Hammer, D., Nickolas, M., & Hughes, P. (2009). Perceiving sacredness in life: Correlates and predictors. *Archive for the Psychology of Religion*, *31*(1), 55–73. https://doi.org/10.1163/157361209x371492

Edelstein, R. S., Newton, N. J., & Stewart, A. J. (2012). Narcissism in midlife: Longitudinal changes in and correlates of women’s narcissistic personality traits. *Journal of Personality*, *80*(5), 1179–1204. https://doi.org/10.1111/j.1467-6494.2011.00755.x

Fang, Y., Niu, Y., & Dong, Y. (2021). Exploring the relationship between narcissism and depression: The mediating roles of perceived social support and life satisfaction. *Personality and Individual Differences*, *173*, Article 110604. https://doi.org/10.1016/j.paid.2020.110604

Forsyth, L., Anglim, J., March, E., & Bilobrk, B. (2021). Dark Tetrad personality traits and the propensity to lie across multiple contexts. *Personality and Individual Differences*, *177*, Article 110792. https://doi.org/10.1016/j.paid.2021.110792

Garcia, D., Adrianson, L., Archer, T., & Rosenberg, P. (2015). The dark side of the affective profiles. *SAGE Open*, *5*(4), Article 215824401561516. https://doi.org/10.1177/2158244015615167

Giacomin, M., & Jordan, C. H. (2016). Self-focused and feeling fine: Assessing state narcissism and its relation to well-being. *Journal of Research in Personality*, *63*, 12–21. https://doi.org/10.1016/j.jrp.2016.04.009

Górnik-Durose, M. E. (2020). Materialism and well-being revisited: The impact of personality. *Journal of Happiness Studies*, *21*(1), 305–326. https://doi.org/10.1007/s10902-019-00089-8

Górnik-Durose, M. E. (2021). Regulatory focus in materialists and its consequences for their well-being. *Journal of Happiness Studies*, *22*(6), 2781–2801. https://doi.org/10.1007/s10902-020-00349-y

Górnik-Durose, M. E., & Pyszkowska, A. (2020). Personality matters – Explaining the link between materialism and well-being in young adults. *Personality and Individual Differences*, *163*, Article 110075. https://doi.org/10.1016/j.paid.2020.110075

Grabovac, B., & Kurbalija, J. Š. (2021). The effects of the dark triad traits on the five pillars of positive psychology: The moderation effect of gender. *Primenjena Psihologija*, *14*(4), 483–508. https://doi.org/10.19090/pp.2021.4.483-508

Hanke, S., Rohmann, E., & Förster, J. (2019). Regulatory focus and regulatory mode – Keys to narcissists’ (lack of) life satisfaction? *Personality and Individual Differences*, *138*, 109–116. https://doi.org/10.1016/j.paid.2018.09.039

Hatchel, T., Negriff, S., & Subrahmanyam, K. (2018). The relation between media multitasking, intensity of use, and well-being in a sample of ethnically diverse emerging adults. *Computers in Human Behavior*, *81*, 115–123. https://doi.org/10.1016/j.chb.2017.12.012

Hernandez, A., & Chalk, H. M. (2021). Social media use: relation to life satisfaction, narcissism, and interpersonal exploitativeness. *Psi Chi Journal of Psychological Research*, *26*(2), 199–207. https://doi.org/10.24839/2325-7342.jn26.2.199

Hewitt, J. M., & Kealy, D. (2022). Pathological narcissism and psychological distress: The mediating effects of vitality, initiative, and mindfulness. *Personality and Individual Differences*, *184*, Article 111185. https://doi.org/10.1016/j.paid.2021.111185

Hill, P. L., & Roberts, B. W. (2012). Narcissism, well-being, and observer-rated personality across the lifespan. *Social Psychological and Personality Science*, *3*(2), 216–223. https://doi.org/10.1177/1948550611415867

Horton, R. S. (2021). Parenthood, subjective well-being, and the moderating effects of parent narcissism. *Journal of Individual Differences*, *42*(2), 57–63. https://doi.org/10.1027/1614-0001/a000329

Huxley, E., Seaton, D. C., & Grenyer, B. F. (2021). Remembered childhood invalidation as a predictor of narcissism, personality functioning, and wellbeing. *Personality and Individual Differences*, *175*, Article 110686. https://doi.org/10.1016/j.paid.2021.110686

Hyun, S., & Ku, X. (2021). Proactive coping mediates the relationship between the narcissism phenotypes and psychological health. *Social Behavior and Personality: An International Journal*, *49*(7), 1–15. https://doi.org/10.2224/sbp.10477

Jankowski, P. J., Hall, E. L., Sandage, S. J., & Dumitrascu, N. (2022). Religious leaders’ well-being: Protective influences for humility and differentiation against narcissism. *Spirituality in Clinical Practice*, *9*(2), 103–113. https://doi.org/10.1037/scp0000265

Joshanloo, M. (2021). Conceptions of happiness mediate the relationship between the dark triad and well-being. *Frontiers in Psychology*, *12*, Article 643351. https://doi.org/10.3389/fpsyg.2021.643351

Juwono, I. D., Kun, B., Demetrovics, Z., & Urbán, R. (2022). Healthy and unhealthy dimensions of perfectionism: Perfectionism and mental health in Hungarian adults. *International Journal of Mental Health and Addiction*. https://doi.org/10.1007/s11469-022-00771-8

Kállay, V. (2020). The investigation of the relationship between narcissism, perfectionism, loneliness, depression, subjective and psychological well-being in a sample of Transylvanian Hungarian and Romanian students. *Studia Universitatis Babeș-Bolyai Psychologia-Paedagogia*, *65*(2), 33–63. https://doi.org/10.24193/subbpsyped.2020.2.02

Kaufman, S. B., & Jauk, E. (2020). Healthy selfishness and pathological altruism: Measuring two paradoxical forms of selfishness. *Frontiers in Psychology*, *11*, Article 1006. https://doi.org/10.3389/fpsyg.2020.01006

Kaufman, S. B., Weiss, B., Miller, J. D., & Campbell, W. K. (2020). Clinical correlates of vulnerable and grandiose narcissism: A personality perspective. *Journal of Personality Disorders*, *34*(1), 107–130. https://doi.org/10.1521/pedi_2018_32_384

Konrath, S., Meier, B. P., & Bushman, B. J. (2014). Development and validation of the single item narcissism scale (SINS). *PLoS ONE*, *9*(8), Article e103469. https://doi.org/10.1371/journal.pone.0103469

Li, R., Yao, M., Chen, Y., & Liu, H. (2020). Parent autonomy support and psychological control, dark triad, and subjective well-being of Chinese adolescents: Synergy of variable- and person-centered approaches. *The Journal of Early Adolescence*, *40*(7), 966–995. https://doi.org/10.1177/0272431619880335

Limone, P., Sinatra, M., & Monacis, L. (2020). Orientations to happiness between the Dark Triad traits and subjective well-being. *Behavioral Sciences*, *10*(5), Article 90. https://doi.org/10.3390/bs10050090

Liu, Y., Zhao, N., & Ma, M. (2021). The Dark Triad traits and the prediction of eudaimonic wellbeing. *Frontiers in Psychology*, *12*, Article 693778. https://doi.org/10.3389/fpsyg.2021.693778

Łojan, A. (2021). Narcissism in the context of depressiveness and happiness. Self-esteem and neuroticism as mediators. *Rozprawy Społeczne*, *15*(1), 20–29. https://doi.org/10.29316/rs/134900

Lopez, M. (2020). *Development and validation of a scale to assess meaning in life: Lopez Meaning Scale (LMS)* (Order No. 13899844). [Doctoral dissertation, Alliant International University]. ProQuest Dissertations & Theses Global.

Luo, Y. L. L., Sedikides, C., & Cai, H. (2020). On the etiology of self-enhancement and its association with psychological well-being. *Social Psychological and Personality Science*, *11*(4), 435–445. https://doi.org/10.1177/1948550619877410

Matshaba, K. L. (2018). *Narcissism, self-esteem and extraversion as predictors of motivations for Facebook use: The impact on subjective-wellbeing.* [Doctoral dissertation, Central China Normal University]. China National Knowledge Infrastructure.

Meng, X., Li, C., Liu, D., & Xu, Y. (2022). The super-short Dark Tetrad: Development and validation within the Chinese context. *Personality and Individual Differences*, *188*, Article 111459. https://doi.org/10.1016/j.paid.2021.111459

Miller, J. D., Dir, A., Gentile, B., Wilson, L., Pryor, L. R., & Campbell, W. K. (2010). Searching for a vulnerable Dark Triad: Comparing factor 2 psychopathy, vulnerable narcissism, and borderline personality disorder. *Journal of Personality*, *78*(5), 1529–1564. https://doi.org/10.1111/j.1467-6494.2010.00660.x

Modersitzki, N., Phan, L. V., Kuper, N., & Rauthmann, J. F. (2021). Who is impacted? Personality predicts individual differences in psychological consequences of the COVID-19 pandemic in Germany. *Social Psychological and Personality Science, 12*(6), 1110–1130. https://doi.org/10.1177/1948550620952576

Monacis, L., Sinatra, M., & Guicciardi, M. (2019). The role of cognitive strategies in the relationships between dark triad personality traits and subje*ctive* well-being. *Psychological research and practice: 15^th^ Days of Applied Psychology,* 95–105. https://doi.org/10.46630/dpp.2020

Morf, C. C., Schürch, E., Küfner, A., Siegrist, P., Vater, A., Back, M., Mestel, R., & Schröder-Abé, M. (2017). Expanding the nomological net of the pathological narcissism inventory: German validation and extension in a clinical inpatient sample. *Assessment*, *24*(4), 419–443. https://doi.org/10.1177/1073191115627010

Musek, J., & Grum, D. K. (2021). The bright side of personality. *Heliyon*, *7*(3), Article e06370. https://doi.org/10.1016/j.heliyon.2021.e06370

Naragon-Gainey, K., & DeMarree, K. G. (2017). Decentering attenuates the associations of negative affect and positive affect with psychopathology. *Clinical Psychological Science*, *5*(6), 1027–1047. https://doi.org/10.1177/2167702617719758

Ng, H. K., Cheung, R. Y. H., & Tam, K. P. (2014). Unraveling the link between narcissism and psychological health: New evidence from coping flexibility. *Personality and Individual Differences*, *70*, 7–10. https://doi.org/10.1016/j.paid.2014.06.006

Peebles, S. A. (2006). *An empirical and existential examination of narcissistic functioning.* [Doctoral dissertation, Auburn University]. ProQuest Dissertations & Theses Global.

Prendergast, C. N., Haahjem Eftedal, N., Fredriksen Ikonomeas, A. G., Brun, A., Huth, H., & Bredesen, M. (2019). The Norwegian version of the five factor narcissism inventory for vulnerable narcissism and the grandiose narcissism subscale of indifference: Psychometric properties of the long‐ and short‐form versions. *Scandinavian Journal of Psychology*, *60*(5), 492–500. https://doi.org/10.1111/sjop.12569

Quang, A. M. T., Van Pham, M., Mai, T. T., Le, G. N. H., & Song, G. A. N. (2022). Self-compassion and students’ well-being among Vietnamese students: Chain mediation effect of narcissism and anxiety. *Journal of Rational-Emotive & Cognitive-Behavior Therapy*, *40*(3), 618–633. https://doi.org/10.1007/s10942-021-00431-1

Rhodewalt, F., Madrian, J. C., & Cheney, S. (1998). Narcissism, self-knowledge organization, and emotional reactivity: The effect of daily experiences on self-esteem and affect. *Personality and Social Psychology Bulletin*, *24*(1), 75–87. https://doi.org/10.1177/0146167298241006

Richardson, K., Hart, W., Tortoriello, G. K., & Breeden, C. J. (2021). An interaction model for the role of self‐evaluations and antagonistic pursuits in subjective well‐being. *British Journal of Psychology*, *112*(2), 493–518. https://doi.org/10.1111/bjop.12473

Rohmann, E., Hanke, S., & Bierhoff, H. W. (2019). Grandiose and vulnerable narcissism in relation to life satisfaction, self-esteem, and self-construal. *Journal of Individual Differences*, *40*(4), 194–203. https://doi.org/10.1027/1614-0001/a000292

Rose, P. (2002). The happy and unhappy faces of narcissism. *Personality and Individual Differences, 33*, 379–391. https://doi.org/10.1016/S0191-8869(01)00162-3

Sakkar Sudha, K., & Shahnawaz, M. G. (2020). Grandiose narcissism and performance in organizations: mediating role of subjective wellbeing. *Leadership, Education, Personality: An Interdisciplinary Journal*, *2*(2), 101–111. https://doi.org/10.1365/s42681-020-00015-0

Sedikides, C., Rudich, E. A., Gregg, A. P., Kumashiro, M., & Rusbult, C. (2004). Are normal narcissists psychologically healthy? Self-esteem matters. *Journal of Personality and Social Psychology, 87*(3), 400–416. [https://doi.org/10.1037/0022-3514.87.3.400](http://dx.doi.org/10.1037/0022-3514.87.3.400)

Sensoy, M. A. (2020). *Social media and mental health: narcissism’s association with well-being is moderated by posting behaviour and received attention in social media.* [Bachelor's thesis, University of Twente]. University of Twente Repository. https://essay.utwente.nl/80436.

Thakur, M. B. (2018). Dark Triad and wellbeing: The Indian context. *Journal of Psychosocial Research.*, *13*(1), 63–72. https://doi.org/10.32381/jpr.2018.13.01.7

Thomaes, S., Stegge, H., Bushman, B. J., Olthof, T., & Denissen, J. (2008). Development and validation of the childhood narcissism scale. *Journal of Personality Assessment*, *90*(4), 382–391. https://doi.org/10.1080/00223890802108162

Tian, Z., Liu, Y., & Teng, Z. (2014). Boredom proneness and narcissism and subject well-being in middle school students, *Chinese Journal of Clinical Psychology*, 22(6), 1062-1067.

Tonna, M., Paglia, F., Ottoni, R., Ossola, P., De Panfilis, C., & Marchesi, C. (2018). Delusional disorder: The role of personality and emotions on delusional ideation. *Comprehensive Psychiatry*, *85*, 78–83. https://doi.org/10.1016/j.comppsych.2018.07.002

Unterrainer, H. F., Ruttinger, J., Lewis, A. J., Anglim, J., Fink, A., & Kapfhammer, H. P. (2016). Vulnerable dark triad personality facets are associated with religious fundamentalist tendencies. *Psychopathology*, *49*(1), 47–52. https://doi.org/10.1159/000443901

Uzarska, A., Czerwiński, S. K., & Atroszko, P. A. (2023). Measurement of shopping addiction and its relationship with personality traits and well-being among Polish undergraduate students. *Current Psychology, 42*, 3794–3810.

https://doi.org/10.1007/s12144-021-01712-9

Van Groningen, A. J., Grawitch, M. J., Lavigne, K. N., & Palmer, S. N. (2021). Every cloud has a silver lining: Narcissism’s buffering impact on the relationship between the Dark Triad and well-being. *Personality and Individual Differences*, *171*, Article 110549. https://doi.org/10.1016/j.paid.2020.110549

Volmer, J., Koch, I. K., & Wolff, C. (2019). Illuminating the ‘dark core’: Mapping global versus specific sources of variance across multiple measures of the dark triad. *Personality and Individual Differences*, *145*, 97–102. https://doi.org/10.1016/j.paid.2019.03.024

Wang, J., Li, T., Wang, K., & Wang, C. (2019). Patience as a mediator between the dark triad and meaning in life. *Applied Research in Quality of Life*, *14*(2), 527–543. https://doi.org/10.1007/s11482-018-9627-y

Wang, X. (2008). *How narcissistic trait influences self-esteem and subjective well-being among college students*. [Master’s thesis, Shaanxi Normal University]. China National Knowledge Infrastructure.

Watson, P. J., Trumpeter, N., O’Leary, B. J., Morris, R. J., & Culhane, S. E. (2006). Narcissism and self-esteem in the presence of imagined others: Supportive versus destructive object representations and the continuum hypothesis. *Imagination, Cognition and Personality*, *25*(3), 253–268. https://doi.org/10.2190/40au-00rd-cv7q-vbpb

Werner, K. M., Smyth, A., & Milyavskaya, M. (2019). Do narcissists benefit from materialistic pursuits? Examining the relation between narcissistic tendencies, extrinsic goals, and well-being. *Collabra: Psychology*, *5*(1). https://doi.org/10.1525/collabra.253

Wink, P., & Dillon, M. (2003). Religiousness, spirituality, and psychosocial functioning in late adulthood: Findings from a longitudinal study. *Psychology and Aging*, *18*(4), 916–924. https://doi.org/10.1037/0882-7974.18.4.916

Womick, J., Atherton, B., & King, L. A. (2020). Lives of significance (and purpose and coherence): Subclinical narcissism, meaning in life, and subjective well-being. *Heliyon*, *6*(5), Article e03982. https://doi.org/10.1016/j.heliyon.2020.e03982

Womick, J., Foltz, R. M., & King, L. A. (2019). “Releasing the beast within”? Authenticity, well-being, and the Dark Tetrad. *Personality and Individual Differences*, *137*, 115–125. https://doi.org/10.1016/j.paid.2018.08.022

Yan, X. (2013). *The Relationship between Personality Traits, Job Satisfaction and Work Engagement on nurses*. [Master’s thesis, The Fourth Military Medical University]. China National Knowledge Infrastructure.

Zajenkowski, M. (2021). How do teenagers perceive their intelligence? Narcissism, intellect, well-being and gender as correlates of self-assessed intelligence among adolescents. *Personality and Individual Differences*, *169*, Article 109978. https://doi.org/10.1016/j.paid.2020.109978

Zajenkowski, M., & Czarna, A. Z. (2015). What makes narcissists unhappy? Subjectively assessed intelligence moderates the relationship between narcissism and psychological well-being. *Personality and Individual Differences, 77*, 50–54. http://dx.doi.org/10.1016/j.paid.2014.12.045

Zajenkowski, M., Czarna, A. Z., Szymaniak, K., & Dufner, M. (2020a). What do highly narcissistic people think and feel about (their) intelligence? *Journal of Personality*, *88*(4), 703–718. https://doi.org/10.1111/jopy.12520

Zajenkowski, M., Leniarska, M., & Jonason, P. K. (2020b). Look how smart I am!: Only narcissistic admiration is associated with inflated reports of intelligence. *Personality and Individual Differences*, *165*, Article 110158. https://doi.org/10.1016/j.paid.2020.110158

Zappala, C. R. (2007). *Well-being:* *The correlation between self-transcendence and psychological and subjective well-being*. [Doctoral dissertation, Institute of Transpersonal Psychology]. ProQuest Dissertations & Theses Global.

Żemojtel-Piotrowska, M. A., Piotrowski, J. P., & Maltby, J. (2017). Agentic and communal narcissism and satisfaction with life: The mediating role of psychological entitlement and self-esteem. *International Journal of Psychology*, *52*(5), 420–424. https://doi.org/10.1002/ijop.12245

Żemojtel-Piotrowska, M., Clinton, A., & Piotrowski, J. (2014). Agentic and communal narcissism and subjective well-being: are narcissistic individuals unhappy? A research report. *Current Issues in Personality Psychology*, *2*(1), 10–16. https://doi.org/10.5114/cipp.2014.43097

Zhang, X. (2011). *Correlation among narcissistic personality, subjective well-being and self-forgiveness*. [Master’s thesis, Guangzhou University]. China National Knowledge Infrastructure.

Zhou, H., Zhang, B., Chen L., & Ye, M. (2009). Development and initial validation of the Narcissistic Personality Questionnaire. *Chinese Journal of Clinical Psychology, 17*(1), 5-7.

Zhu, C., Su, R., Zhang, X., & Liu, Y. (2021). Relation between narcissism and meaning in life: the role of conspicuous consumption. *Heliyon*, *7*(9), Article e07885. https://doi.org/10.1016/j.heliyon.2021.e07885

Zhu, Y. (2016). The relationship between the dark triad and subjective well-being among college students. *Journal of Heilongjiang Vocational Institute of Ecological Engineering, 29*(1), 64–66. http://doi.org/10.3969/j.issn.1674-6341.2016.01.027

Zondag, H. (2005). Between imposing one’s will and protecting oneself. Narcissism and the meaning of life among Dutch pastors. *Journal of Religion and Health*, *44*(4), 413–426. https://doi.org/10.1007/s10943-005-7180-0

Zondag, H. J., van Halen, C., & Wojtkowiak, J. (2009). Overt and covert narcissism in Poland and The Netherlands. *Psychological Reports*, *104*(3), 833–843. https://doi.org/10.2466/pr0.104.3.833-843

Zuckerman, M., & O’Loughlin, R. E. (2009). Narcissism and well-being: A longitudinal perspective. *European Journal of Social Psychology, 39*(6), 957–972. https://doi.org/10.1002/ejsp.594

1. **OVERVIEW OF INCLUDED STUDIES**

| **Study** | **Sample**  **size** | **Age**  **Mean** | **Male** | **Paper**  **Language** | **Country** | **Individualism** | **Published**  **status** | **Grandiose**  **Narcissism** | **Vulnerable**  **narcissism** | **Hedonism** | **Eudaemonia** | **Mixed** |
| --- | --- | --- | --- | --- | --- | --- | --- | --- | --- | --- | --- | --- |
| Adeeb et al., 2020 | 233 | 18.2 |  | English | Pakistan | 14 | Published | 1X |  | 1X |  |  |
| Aghababaei & Błachnio, 2015 | 220 | 19.69 | 8.2 | English | Poland | 60 | Published | 3X |  | 2X | 1X |  |
| Aghababaei et al., 2014 | 223 | 31.24 | 40.36 | English | Iran | 41 | Published | 1X |  | 1X |  |  |
| Akinci, 2015 | 559 | 33.46 | 39 | English | Turkey | 37 | Unpublished | 1X | 1X | 2X |  |  |
| Aminnuddin, 2020 | 277 | 19.62 | 61.73 | English | Brunei |  | Published | 3X |  | 2X | 1X |  |
| Anderson & Costello, 2009 | 100 |  |  | English | USΑ | 91 | Unpublished | 1X |  | 1X |  |  |
| Atroszko et al., 2018 | 1157 | 20.33 | 47.2 | English | Poland | 60 | Published | 1X |  | 1X |  |  |
| Ben Shlomo & Taubman-Ben-Ari, 2017 | 108 | 57.7 | 100 | English | Israel | 54 | Published | 1X |  | 1X |  |  |
| Błachnio & Przepiórka, 2018 | 360 | 22.22 | 36 | English | Poland | 60 | Published | 1X |  | 1X |  |  |
| Bogart et al., 2004 | 109 | 18.87 | 25 | English | USΑ | 91 | Published | 1X |  | 1X |  |  |
| Bourbonnais & Durand, 2018 | 541 | 25.98 | 45 | English | Global^a^ | - | Published | 1X |  | 1X |  |  |
| Brailovskaia & Margraf, 2016, Sample 1 | 155 | 25.28 | 36.13 | English | Germany | 67 | Published | 2X |  | 2X |  |  |
| Brailovskaia & Margraf, 2016, Sample 2 | 790 | 23.42 | 28.86 | English | Germany | 67 | Published | 2X |  | 2X |  |  |
| Brailovskaia & Margraf, 2019, Sample 1 | 438 | 21.58 | 34 | English | Germany | 67 | Published | 2X |  | 2X |  |  |
| Brailovskaia & Margraf, 2019, Sample 2 | 82 | 22.7 | 19.51 | English | Germany | 67 | Published | 3X |  | 3X |  |  |
| Brailovskaia & Margraf, 2019, Sample 3 | 328 | 24.83 | 33.8 | English | Germany | 67 | Published | 1X |  | 1X |  |  |
| Brunell & Buelow, 2019, Sample 1 | 145 |  |  | English | USΑ | 91 | Published | 4X | 2X | 6X |  |  |
| Brunell & Buelow, 2019, Sample 2 | 146 |  |  | English | USΑ | 91 | Published | 4X | 2X | 6X |  |  |
| Brunell & Buelow, 2019, Sample 3 | 145 |  |  | English | USΑ | 91 | Published | 4X | 2X | 6X |  |  |
| Brunell & Buelow, 2019, Sample 4 | 436 | 19.09 | 42.2 | English | USΑ | 91 | Published | 4X | 2X | 6X |  |  |
| Cai et al., 2017 | 2828 | 15.6 | 44.02 | Chinese | China | 20 | Published | 2X |  | 1X | 1X |  |
| Çevik et al., 2021 | 461 | 11.51 | 100 | English | Turkey | 37 | Published | 1X |  | 1X |  |  |
| Chamberlain & Haaga, 2001 | 107 | 48.9 | 42.99 | English | USΑ | 91 | Published | 2X |  | 2X |  |  |
| Charzyńska et al., 2021 | 1157 | 20.33 | 47.2 | English | Poland | 60 | Published | 1X |  | 1X |  |  |
| Clark et al., 2010 | 322 |  | 27 | English | USΑ | 91 | Published | 1X |  | 1X |  |  |
| Ding et al., 2016 | 303 | 21.5 | 39.93 | Chinese | China | 20 | Published | 1X |  | 1X |  |  |
| Doehring et al., 2009 | 113 | 56.1 | 60 | English | USΑ | 91 | Published | 1X |  |  | 1X |  |
| Edelstein et al., 2012 | 70 | 53 | 0 | English | USΑ | 91 | Published | 8X | 4X | 6X | 6X |  |
| Fang et al., 2021 | 831 | 19.57 | 44.28 | English | China | 20 | Published | 1X |  | 1X |  |  |
| Forsyth et al., 2021 | 615 | 26.83 | 37.1 | English | Australia | 90 | Published | 1X |  | 1X |  |  |
| Garcia et al., 2015 | 1000 | 31.5 | 66.7 | English | USΑ | 91 | Published | 1X |  | 1X |  |  |
| Giacomin & Jordan, 2016 | 147 | 18.61 | 20.41 | English | Canada | 80 | Published | 10X | 4X | 14X |  |  |
| Gornik-Durose, 2020, Sample 1 cross sectional | 123 | 35.07 | 26.8 | English | Poland | 60 | Published | 1X | 1X | 2X |  |  |
| Gornik-Durose, 2020， Sample 1 longitudinal | 123 | 35.07 | 26.8 | English | Poland | 60 | Published | 2X | 2X | 4X |  |  |
| Gornik-Durose, 2020, Sample 2 | 360 | 35.68 | 32.5 | English | Poland | 60 | Published | 1X | 1X |  |  | 2X |
| Górnik-Durose, 2021 | 287 | 24.6 | 19.5 | English | Poland | 60 | Published | 2X |  | 2X |  |  |
| Grabovac & Kurbalija, 2021 | 439 | 26.07 | 46 | English | Serbia | 25 | Published | 1X |  |  |  | 1X |
| Górnik-Durose & Pyszkowska, 2020 | 415 | 22.15 | 16.9 | English | Poland | 60 | Published | 2X |  | 2X |  |  |
| Hanke et al., 2019, Sample 1 | 297 | 23.31 | 22.22 | English | Germany | 67 | Published | 2X | 2X | 4X |  |  |
| Hanke et al., 2019, Sample 2 | 143 | 26.01 | 27.97 | English | Germany | 67 | Published | 2X | 2X | 4X |  |  |
| Hatchel et al., 2018 | 263 | 20.58 |  | English | USΑ | 91 | Published | 1X |  | 1X |  |  |
| Hernandez & Chalk, 2021 | 2016 | 20.26 | 25 | English | USΑ | 91 | Published | 1X |  | 1X |  |  |
| Hewitt & Kealy, 2022 | 119 | 20.8 | 25.2 | English | Canada | 80 | Published | 1X | 1X |  | 2X |  |
| Hill & Roberts, 2012, Sample 1 | 798 | 37.73 | 35 | English | USΑ | 91 | Published | 1X |  | 1X |  |  |
| Hill & Roberts, 2012, Sample 2 | 449 |  |  | English | USΑ | 91 | Published | 1X |  | 1X |  |  |
| Hill & Roberts, 2012, Sample 3 | 179 |  |  | English | USΑ | 91 | Published | 1X |  | 1X |  |  |
| Hill & Roberts, 2012, Sample 4 | 161 |  |  | English | USΑ | 91 | Published | 1X |  | 1X |  |  |
| Hill & Roberts, 2012, Sample 5 | 339 |  |  | English | USΑ | 91 | Published | 1X |  | 1X |  |  |
| Horton, 2021 | 388 |  | 52 | English | USΑ | 91 | Published | 4X | 2X | 3X | 3X |  |
| Huxley et al., 2021 | 291 | 27.61 | 18.2 | English | Australia | 90 | Published | 3X | 1X | 4X |  |  |
| Hyun & Ku, 2021 | 280 | 22.99 | 28.21 | English | South Korea | 18 | Published | 1X | 1X | 2X |  |  |
| Jankowski et al., 2022 | 75 | 34.97 | 70.7 | English | USA | 91 | Published |  | 1X |  |  | 1X |
| Joshanloo, 2021 | 1177 | 40.96 | 48.9 | English | South Korea | 18 | Published | 1X |  |  |  | 1X |
| Juwono et al., 2022 | 4340 |  | 49.3 | English | Hungary | 80 | Published | 1X |  |  | 1X |  |
| Kállay, 2020 | 535 | 20.01 | 22.64 | English | Romania | 30 | Published | 5X |  |  | 5X |  |
| Kaufman & Jauk, 2020 | 891 | 37.12 | 46.8 | English | USA | 91 | Published | 6X |  | 3X | 3X |  |
| Kaufman et al., 2020 | 388 | 35 | 54 | English | USA | 91 | Published | 1X | 1X | 2X |  |  |
| Konrath et al., 2014 | 110 | 19.7 | 40 | English | USA | 91 | Published | 2X |  | 2X |  |  |
| Li et al., 2020 | 1533 | 15.29 | 44.94 | English | China | 20 | Published | 2X |  | 2X |  |  |
| Limone et al., 2020 | 460 | 22.17 | 59.35 | English | Italy | 76 | Published | 1X |  | 1X |  |  |
| Liu et al., 2021 | 705 | 25.46 | 39.4 | English | China | 20 | Published | 2X |  | 1X | 1X |  |
| Łojan, 2021 | 122 | 25.7 | 53 | English | Poland | 60 | Published | 1X | 1X | 2X |  |  |
| Lopez, 2020 | 329 | 38.5 | 61.1 | English | USA | 91 | Unpublished | 5X |  | 2X | 3X |  |
| Luo et al., 2020 | 608 | 18.29 | 44.1 | English | China | 20 | Published | 6X |  | 6X |  |  |
| Matshaba, 2018 | 627 |  |  | English | Botswana |  | Unpublished | 2X |  | 2X |  |  |
| Meng et al., 2022 | 431 | 38.77 | 37.35 | English | China | 20 | Published | 1X |  | 1X |  |  |
| Miller et al., 2010 | 361 | 19.1 | 37.4 | English | Greece | 35 | Published | 1X | 1X | 2X |  |  |
| Modersitzki et al., 2021 | 1320 | 44.36 | 41.36 | English | Germany | 67 | Published | 2X |  | 2X |  |  |
| Monacis et al., 2019 | 460 | 42.17 | 59.35 | English | Italy | 76 | Unpublished | 2X |  | 2X |  |  |
| Morf et al., 2017 | 246 | 26.78 | 32.5 | English | Switzerland & Germany | 68 | Published | 6X | 4X | 10X |  |  |
| Musek & Grum, 2021 | 495 | 33.8 | 24.44 | English | Slovenia | 27 | Published | 1X |  | 1X |  |  |
| Naragon-Gainey & DeMarree, 2017 | 568 | 19.25 | 52.64 | English | USA | 91 | Published | 1X | 1X | 2X |  |  |
| Ng et al., 2014 | 179 | 21.02 | 55.87 | English | Hongkong | 25 | Published | 2X | 2X | 4X |  |  |
| Peebles, 2006 | 190 | 22 | 35.8 | English | USA | 91 | Unpublished | 2X |  |  | 2X |  |
| Prendergast et al., 2019 | 214 | 26.8 | 33.7 | English | Norway | 69 | Published | 1X | 1X |  | 2X |  |
| Quang et al., 2022 | 420 | 18.89 |  | English | Vietnam | 20 | Published | 1X |  |  | 1X |  |
| Rhodewalt et al., 1998, Sample 1 | 49 |  | 43 | English | USA | 91 | Published | 1X |  | 1X |  |  |
| Rhodewalt et al., 1998, Sample 2 | 79 |  | 42 | English | USA | 91 | Published | 1X |  | 1X |  |  |
| Richardson et al., 2021, Sample 1 | 417 | 37.02 | 46.28 | English | USA | 91 | Published | 1X |  | 1X |  |  |
| Richardson et al., 2021, Sample 2 | 450 | 35.53 | 46.89 | English | USA | 91 | Published | 2X |  | 2X |  |  |
| Rohmann et al., 2019 | 253 | 30.6 | 23.7 | English | Germany |  | Published | 1X | 1X | 2X |  |  |
| Rose, 2002 | 262 |  | 44.28 | English | USA | 91 | Published | 8X | 8X | 16X |  |  |
| Sakkar Sudha & Shahnawaz, 2020 | 293 | 37.3 | 50.51 | English | India | 48 | Published | 2X |  | 2X |  |  |
| Sedikides et al., 2004, Sample 1 | 604 |  |  | English | USA | 91 | Published | 1X |  | 1X |  |  |
| Sedikides et al., 2004, Sample 2 | 149 |  | 28.19 | English | USA | 91 | Published | 4X |  | 4X |  |  |
| Sedikides et al., 2004, Sample 3 | 81 |  | 25.93 | English | USA | 91 | Published | 2X |  | 2X |  |  |
| Sedikides et al., 2004, Sample 4 | 158 | 34.11 | 50 | English | USA | 91 | Published | 4X |  | 4X |  |  |
| Sedikides et al., 2004, Sample 5 | 154 |  | 31.82 | English | USA | 91 | Published | 4X |  | 4X |  |  |
| Sedikides et al., 2004, Sample 6 | 155 |  | 15.48 | English | USA | 91 | Published | 1X |  | 1X |  |  |
| Sensoy, 2020 | 136 | 20.78 | 21.32 | English | Netherlands | 80 | Unpublished | 1X |  | 1X |  |  |
| Thakur, 2018 | 97 | 19.18 | 14.91 | English | India | 48 | Published | 3X |  | 2X | 1X |  |
| Thomaes et al., 2008 | 238 | 11.5 | 47 | English | Netherlands | 80 | Published | 1X |  | 1X |  |  |
| Tian et al., 2014 | 472 |  | 47.67 | Chinese | China | 20 | Published | 1X | 1X |  |  | 2X |
| Tonna et al., 2018 | 91 | 48.8 | 34.1 | English | USA | 91 | Published | 1X | 1X | 2X |  |  |
| Unterrainer et al., 2016 | 327 | 25.1 | 29.1 | English | Germany | 67 | Published |  | 3X |  | 3X |  |
| Uzarska et al., 2023 | 1156 | 20.33 | 47.2 | English | Poland | 60 | Published | 1X |  | 1X |  |  |
| Van Groningen et al., 2021, Sample 1 | 435 |  |  | English | USA | 91 | Published | 1X |  | 1X |  |  |
| Van Groningen et al., 2021, Sample 2 | 515 |  |  | English | USA | 91 | Published | 1X | 1X | 2X |  |  |
| Volmer et al.., 2019 | 395 | 25.15 | 23 | English | Germany | 67 | Published | 3X |  | 3X |  |  |
| Wang et al., 2019 | 434 | 30.47 | 22 | English | China | 20 | Published | 1X |  |  | 1X |  |
| Wang, 2008 | 406 | 19.42 | 37.44 | Chinese | China | 20 | Unpublished | 2X | 2X |  |  | 4X |
| Watson et al., 2006 | 189 | 19.5 | 33.24 | English | USA | 91 | Published | 4X |  | 4X |  |  |
| Werner et al., 2019 | 576 |  |  | English | Canada | 80 | Published | 3X |  | 2X | 1X |  |
| Wink & Dillon, 2003 | 122 |  | 47 | English | USA | 91 | Published | 1X |  |  | 1X |  |
| Womick et al., 2020 | 415 | 34.5 | 50.5 | English | USA | 91 | Published | 1X |  | 1X |  |  |
| Womick et al., 2019, Sample 1 | 404 | 35.7 | 50.1 | English | USA | 91 | Published | 4X |  | 2X | 1X | 1X |
| Womick et al., 2019, Sample 2 | 415 | 34.5 | 50.5 | English | USA | 91 | Published | 4X |  | 2X | 1X | 1X |
| Yan, 2013 | 501 | 19.9 | 0 | Chinese | China | 20 | Unpublished | 1X |  | 1X |  |  |
| Zajenkowski, 2021 | 428 | 15.89 | 21.5 | English | Poland | 60 | Published | 1X |  | 1X |  |  |
| Zajenkowski & Czarna, 2015, Sample 1 | 202 | 23.03 | 43.56 | English | Poland | 60 | Published | 1X |  | 1X |  |  |
| Zajenkowski & Czarna, 2015, sample 2 | 154 | 23.1 | 26.62 | English | Poland | 60 | Published | 1X |  | 1X |  |  |
| Zajenkowski et al., 2020a | 306 | 24.07 | 30.39 | English | Poland | 60 | Published | 1X | 1X | 2X |  |  |
| Zajenkowski et al., 2020b | 311 | 23.47 | 33.44 | English | Poland | 60 | Published | 1X |  | 1X |  |  |
|  |  |  |  |  |  |  |  |  |  |  |  |  |
| Zappala, 2007 | 63 |  | 19.05 | English | USA/Germany | 79 | Unpublished |  | 2X | 1X | 1X |  |
| Żemojtel‐Piotrowska et al., 2014 | 138 | 21.57 | 47.83 | English | Poland | 60 | Published | 4X |  | 4X |  |  |
| Żemojtel‐Piotrowska et al., 2017, Sample 1 | 137 | 21.56 | 47.4 | English | Poland | 60 | Published | 3X |  | 3X |  |  |
| Żemojtel‐Piotrowska et al., 2017, Sample 2 | 111 | 20.07 | 26 | English | UK | 89 | Published | 2X |  | 2X |  |  |
| Zhang, 2011 | 714 | 21 | 39.88 | Chinese | China | 20 | Unpublished | 3X | 3X | 6X |  |  |
| Zhou et al., 2009 | 143 |  | 30.77 | Chinese | China | 20 | Published | 1X |  | 1X |  |  |
| Zhu et al., 2021 | 480 | 19.7 | 48.13 | English | China | 20 | Published | 1X | 1X |  | 2X |  |
| Zhu, 2016, Sample 1 | 190 | 19.9 | 100 | Chinese | China | 20 | Published | 1X |  | 1X |  |  |
| Zhu, 2016, Sample 2 | 241 | 19.9 | 0 | Chinese | China | 20 | Published | 1X |  | 1X |  |  |
| Zondag, 2005 | 196 | 50 | 69 | English | Netherlands | 80 | Published | 2X | 2X |  | 4X |  |
| Zondag et al., 2009, Sample 1 | 156 | 21.2 | 25.15 | English | Poland | 60 | Published | 2X | 2X |  | 4X |  |
| Zondag et al., 2009, Sample 2 | 167 | 24.5 | 14.1 | English | Netherlands | 80 | Published | 2X | 2X |  | 4X |  |
| Zuckerman & O’Loughlin, 2009 | 176 |  | 27.84 | English | USA | 91 | Published | 7X |  |  |  | 7X |

Note. ^a^The sample from Bourbonnais & Durand (2018) did not report specific country information. The majority of participants were located in North America (61%) or Europe (26%). Therefore, we excluded this effect size from the moderation analysis testing the effect of individualism, but we included it in other analyses.

1. **CATEGORIZATION OF NARCISSISM MEASURES**

| **Measures** | **Measure Reference** | **Included Study** | **Number of Items** | **Sample Item** | **Grandiose narcissism** | **Vulnerable narcissism** | |
| --- | --- | --- | --- | --- | --- | --- | --- |
| Narcissistic Personality Inventory (NPI-13) | Gentile et al., 2013 | Brailovskaia et al., 2019; Brailovskaia & Margraf, 2016; Żemojtel‐Piotrowska et al., 2017 | 13 | “I find it easy to manipulate people.” | X | |  |
| Narcissistic Personality Inventory (NPI-16) | Ames et al., 2006 | Adeeb et al., 2020; Akinci, 2015; Anderson & Costello, 2009; Giacomin & Jordan, 2016; Hatchel et al., 2018; Kaitlyn et al., 2019; Kállay, 2020; Wang, 2008; Sakkar Sudha & Shahnawaz, 2020; Yan, 2013 | 16 | “I really like to be the center of attention.” | X | |  |
| Six grandiose items from Narcissism Personality Inventory-16 | Barelds & Dijkstra, 2010 | Matshaba, 2018 | 6 | “I know that I am good because everybody keeps telling me so” | X | |  |
| Narcissistic Personality Inventory (NPI -23) | Corry et al., 2008 | Ng et al., 2014 | 23 | Leadership/authority (e.g., “I have a strong will to power”) and exhibitionism/entitlement (e.g., “I will never be satisfied until I get all that I deserve”). | X | |  |
| Narcissistic Personality Inventory (NPI-29) | Raskin & Hall, 1979 | Chamberlain & Haaga, 2001 | 29 |  | X | |  |
| Narcissistic Personality Inventory (NPI-34) | Bazińska & Drat-Ruszczak, 2000 | Błachnio & Przepiórka, 2018; Górnik-Durose, 2020; Górnik-Durose, 2021; Łojan, 2021; Zajenkowski, 2021; Zajenkowski et al., 2020b | 34 | “I like being in the center of attention” | X | |  |
| Narcissistic Personality Inventory (NPI-37) | Emmons, 1987 | Bogart et al., 2004; Rhodewalt et al., 1998; Rose, 2002 | 37 | “I know that I am good because everyone keeps telling me so” | X | |  |
| Adaptive Narcissism factor from Narcissistic Personality Inventory (NPI-40) | Raskin & Terry, 1988 | Watson et al., 2005 |  |  | X | |  |
| Maladaptive Narcissism factor from Narcissistic Personality Inventory (NPI-40) | Raskin & Terry, 1988 | Watson et al., 2005 |  |  | X | |  |
| Narcissistic Personality Inventory (NPI-40) | Raskin & Terry, 1988 | Ben Shlomo & Taubman-Ben-Ari, 2017; Brailovskaia & Margraf, 2019; Brunell & Buelow, 2019; Clark et al., 2010; Hanke et al., 2019; Hill & Roberts, 2012; Horton, 2021; Konrath et al., 2014; Luo et al., 2020; Miller et al., 2010; Morf et al., 2017; Nickolas et al., 2019; Richardson et al., 2021; Rohmann et al., 2019; Sedikides et al., 2004; Volmer et al., 2019; Womick et al., 2019; Womick et al., 2020; Zuckerman & O' Loughlin, 2009 | 40 | Each item includes a pair of statements, one narcissistic (e.g., “I have a natural talent for influencing people”) and one non-narcissistic (e.g., “I am not good at influencing people”). | X | |  |
| Narcissistic Personality Inventory (NPI-54) | Bazińska & Drat- Ruszczak, 2000; Raskin & Terry, 1988 | Peebles, 2007; Sedikides et al., 2004; Żemojtel-Piotrowska et al., 2014 | 54 |  | X | |  |
| Grandiosity subscale of Pathological Narcissism Inventory (PNI) | Pincus et al., 2009 | Brunell & Buelow, 2019; Giacomin & Jordan, 2016; Horton, 2021; Hyun & Ku, 2021; Tonna et al., 2018; Unterrainer et al., 2016 |  | Grandiose fantasy (e.g., “I often fantasize about being admired and respected”), exploitative (e.g., “I find it easy to manipulate people”), and self-sacrificing self-enhancement (e.g., “I try to show what a good person I am through my sacrifices”) and entitlement rage (e.g., “I get angry when criticized”). | X | |  |
| Vulnerable subscale of Pathological Narcissism Inventory (PNI) | Pincus et al., 2009 | Brunell & Buelow, 2019; Giacomin & Jordan, 2016; Horton, 2021; Hyun & Ku, 2021; Tonna et al., 2018; Unterrainer et al., 2016 |  | Contingent self-esteem (e.g., “I need others to acknowledge me”), devaluing (e.g., “Sometimes I avoid people because I’m concerned that they’ll disappoint me”) |  | | X |
| The Brief Pathological Narcissism Inventory (B-PNI) | Schoenleber et al., 2015 | Jankowski et al., 2022 | 28 |  |  | | X |
| Grandiose subscale of Brief Pathological Narcissism Inventory (B-PNI) | Schoenleber et al., 2015 | Brunell & Buelow, 2019; Huxley et al., 2021 | 12 |  | X | |  |
| Vulnerable subscale of Brief Pathological Narcissism Inventory (B-PNI) | Schoenleber et al., 2015 | Brunell & Buelow, 2019; Huxley et al., 2021 | 16 |  |  | | X |
| Grandiose subscale of Super Brief Pathological Narcissism Inventory (SB-PNI) | Schoenleber et al., 2015 | Hewitt & Kealy, 2022; Naragon-Gainey & DeMarree, 2017 | 6 |  | X | |  |
| Vulnerable subscale of Super Brief Pathological Narcissism Inventory (SB-PNI) | Schoenleber et al., 2015 | Hewitt & Kealy, 2022; Naragon-Gainey & DeMarree, 2017 | 6 |  |  | | X |
| Grandiose narcissism subscale of Five Factor Narcissism Inventory (FFNI) | Glover et al., 2012 | Kaufman & Jauk, 2020 |  |  | X | |  |
| Vulnerable narcissism subscale of Five Factor Narcissism Inventory (FFNI) | Glover et al., 2012 | Kaufman & Jauk, 2020 |  |  |  | | X |
| Grandiose narcissism subscale of Five Factor Narcissism Inventory Short Form (FFNI-SF) | Sherman et al., 2015 | Kaufman et al., 2020 | 60 |  | X | |  |
| Vulnerable narcissism subscale of Five Factor Narcissism Inventory Short Form (FFNI-SF) | Sherman et al., 2015 | Kaufman et al., 2020 |  |  |  | | X |
| Vulnerable narcissism subscale of Five Factor Narcissism Inventory Short Form (FFNI-SF) - Norwegian version | Prendergast et al., 2019 | Prendergast et al., 2019 | 16 |  |  | | X |
| Indifference subscale of Grandiose Narcissism from Five Factor Narcissism Inventory Short Form (FFNI-SF) | Prendergast et al., 2019 | Prendergast et al., 2019 | 10 |  | X | |  |
| Dirty Dozen measure of the Dark Triad (DTDD) | Jonason & Webster, 2010 | Aghababaei et al., 2014; Garcia et al., 2015; Garcia et al., 2015; Liu et al., 2021; Musek & Grum, 2021; Thakur, 2018; Thakur, 2018; Volmer et al., 2019; Wang et al., 2019; Zajenkowski & Czarna, 2015; Zhu, 2016 | 4 | “I tend to want others to pay attention to me.” | X | |  |
| Narcissism subscale of Short Dark Triads (SD3) | Jones & Paulhus, 2014 | Aghababaei & Błachnio, 2015; Aminnuddin, 2020; Bourbonnais & Durand, 2018; Cai et al., 2017; Forsyth et al., 2021; Grabovac & Kurbalija, 2021; Li et al., 2020; Monacis et al., 2019; Volmer et al., 2019; Womick et al., 2019 | 9 | “I tend to want others to admire me” | X | |  |
| Narcissism subscale of super short Dark Tetrad Scale (SD4) | Meng et al., 2022 | Meng et al., 2022 | 4 |  | X | |  |
| Narcissistic Grandiosity Scale (NGS) | Rosenthal et al., 2007 | Brunell & Buelow, 2019; Huxleya et al., 2021; Luo et al., 2020; Van Groningen et al., 2021 | 16 | The Narcissistic Grandiosity Scale (NGS) consists of 16 adjectives: advanced, admirable, brave, enviable, excellent, extraordinary, honorable, omnipotent, outstanding, perfect, powerful, prestigious, respectable, talented, unique, and vigorous | X | |  |
| Narcissistic Inventory-Revised (NI-R) | Rohmann et al., 2012 | Hanke et al., 2019; Rohmann et al., 2019 | 36 |  |  | | X |
| Admiration subscale of Narcissistic Admiration and Rivalry Questionnaire (NARQ) | Back et al., 2013 | Zajenkowsk et al., 2020;  Fang et al., 2021 | 9 | “I show others how special I am.” | X | |  |
| Rivalry subscale of Narcissistic Admiration and Rivalry Questionnaire (NARQ) | Back et al., 2013 | Zajenkowsk et al., 2020;  Fang et al., 2021 | 9 | “I secretly take pleasure in the failure of my rivals.” | X | |  |
| Narcissistic Admiration and Rivalry Questionnaire short form (NARQ-S) | Leckelt et al., 2017 | Juwono et al., 2022 | 6 | “I deserve to be seen as a great personality.”\| | X | |  |
| Autonomy subscale from the California Adult Q-Sort (CAQ). | Wink, 1992 | Edelstein et al., 2012 | 11 |  | X | |  |
| Hypersensitivity subscale from the California Adult Q-Sort (CAQ). | Wink, 1992 | Edelstein et al., 2012 | 12 |  |  | | X |
| Willfulness subscale from the California Adult Q-Sort (CAQ). | Wink, 1992 | Edelstein et al., 2012 | 10 |  | X | |  |
| Centrifugal dimension of Nederlandstalige Narcisme Schaal (NNS, Dutch-language narcissism scale) | Ettema & Zondag, 2002 | Zondag, 2005 | 11 | “I can easily get others to do what I feel is necessary’’ (centrifugal)” | X | |  |
| Centripetal dimension of Nederlandstalige Narcisme Schaal (NNS, Dutch-language narcissism scale) | Ettema & Zondag, 2002 | Zondag, 2005 | 8 | “When I enter a room I am often painfully aware of the way others look at me” |  | | X |
| Overt subscale of Dutch Narcissism Scale | Ettema & Zondag, 2002 | Zondag et al., 2009 | 9 | “I can easily get others to do what I feel is necessary” | X | |  |
| Covert subscale of Dutch Narcissism Scale | Ettema & Zondag, 2002 | Zondag et al., 2009 | 17 | “When I enter a room I am often painfully aware of the way others look at me.” |  | | X |
| Childhood Narcissism Scale (CNS) | Thomaes et al., 2008 | Çevik et al., 2021; Thomaes et al., 2008 | 10 | “I am a very special person” | X | |  |
| Communal Narcissism Inventory (CNI) | Gebauer et al., 2012 | Brunell & Buelow, 2019; Kaufman & Jauk, 2020; Luo et al., 2020; Żemojtel-Piotrowska et al., 2014; Żemojtel-Piotrowska et al., 2017 | 16 | “I am the most helpful person I know” | X | |  |
| Covert narcissism subscale of The Narcissism Scale (Chinese) | Zheng & Huang, 2005 | Ding et al., 2016; Tian et al., 2014; Zhang, 2011; Zhu et al., 2021 | 20 | “People and things around me often make me feel very dissatisfied.” |  | | X |
| Overt narcissism subscale of The Narcissism Scale (Chinese) | Zheng & Huang, 2005 | Ding et al., 2016; Tian et al., 2014; Zhang, 2011; Zhu et al., 2021 | 15 | “I know I am excellent, because everyone says I am born with a talent for leadership.” | X | |  |
| CPI’s Narcissism scale | Wink & Gough, 1990 | Wink & Dillon, 2003 | 49 |  | X | |  |
| Hypersensitive Narcissism Scale (HSNS) | Hendin & Cheek, 1997 | Akinci, 2015; Brunell & Buelow, 2019; Giacomin & Jordan, 2016; Gornik-Durose, 2020; Miller et al., 2010; Ng et al., 2014; Van Groningen et al., 2021; Wang, 2008; Wang, 2008; Zajenkowski et al., 2020a; Zappala, 2008 | 10 | “I often interpret the remarks of others in a personal way.” |  | | X |
| Hypersensitive Narcissism Scale (HSNS) & four subscales of the Pathological Narcissism Inventory (Contingent Self-Esteem, Hiding the Self, Devaluating, & Entitled Rage) | Miller et al., 2020 | Miller et al., 2020 |  |  |  | | X |
| Chinese Narcissistic Personality Questionnaire | Zhou et al., 2009 | Zhou et al., 2009 | 34 |  | X | |  |
| Vietnamese Narcissism Personality Inventory | Ninh, 2020; Raskin & Terry, 1988 | Quang, 2022 | 22 |  | X | |  |
| Vulnerable Narcissism Scale | Pilch & GórnikDurose, 2017 | Łojan, 2021 | 10 | “I don’t like sharing my merits with others.” |  | | X |
| Raskin and Novacek Narcissism Scale (RNNS) | Raskin & Novacek, 1989 | Rose, 2002 | 42 | “I am an important person” | X | |  |
| Narcissistic Personality Disorder Scale (NPDS) | Ashby et al., 1979 | Rose, 2002 | 17 | “I have felt embarrassed over the type of work that one or more of my family members have done” |  | | X |
| Serkownek Narcissism Scale (SNS) | Serkownek, 1975 | Rose, 2002 | 18 | “I frequently find myself worrying about something” |  | | X |
| Naricissism subscale of Naughty Nine measure of Dark Triad | Küfner et al., 2015 | Modersitzki et al., 2021 |  |  | X | |  |
| Self-rating on 11 adjectives - state | Giacomin & Jordan, 2016 | Giacomin & Jordan, 2016 |  | Participants indicated the extent to which 11 adjectives described their behavior: egotistical, self-focused, vain, manipulative, attention seeking, arrogant, narcissistic, self-centered, conceited, self-indulgent, selfish | X | |  |
| Single Item Narcissism Scale (SINS) | Konrath et al., 2014 | Atroszko et al., 2018; EdytaCharzyńska et al., 2021; Giacomin & Jordan, 2016; Konrath et al., 2014; Uzarska et al., 2023 | 1 | “To what extent do you agree with this statement: I am a narcissist. (Note: The word ‘narcissist’ means egotistical, self-focused, and vain).” | X | |  |
| Psychological Entitlement Scale (PES) | Campbell et al., 2004 | Huxley et al., 2021 | 9 | “I deserve more things in my life” | X | |  |

1. **CATEGORIZATION OF WELLBEING MEASURES**

| **Measures** | **Measure Reference** | **Included study** | **Number of items** | **Sample items** | **Hedonism** | **Eudaemonia** | **Mixed** |
| --- | --- | --- | --- | --- | --- | --- | --- |
| Affect Balance | Richter et al., 2017 | Modersitzki et al., 2021 | 2 |  | X |  |  |
| Affect Balance Scale (ABS) | Bradburn, 1969 | Górnik-Durose, 2021; Górnik-Durose & Pyszkowska, 2020; Sedikides et al., 2004 | 10 |  | X |  |  |
| Positive affect subscale of Affect Balance Scale (ABS) | Bradburn, 1969 | Li et al., 2020 | 8 | “I am optimistic about the future” | X |  |  |
| Affective Wellbeing Scale (AWBS) | Diener et al., 2010 | Luo et al., 2020 | 8 | Participants rated the frequency with which they experienced negative (unpleasant, sad, angry, afraid) and positive (happy, pleasant, joyful, contented) affect. | X |  |  |
| Campbell et al. scale | Campbell et al., 1976 | Sedikides et al., 2004 | 10 | Describe your present life by circling a number for each of the following scales (e.g., “boring-interesting”; disappointing rewarding” ) | X |  |  |
| Positive Affect | Forsyth et al., 2021 | Forsyth et al., 2021 | 5 | Joy, happiness, amusement, satisfaction, enjoyment | X |  |  |
| Positive Affect | Womick et al., 2019 | Womick et al., 2019 | 4 |  | X |  |  |
| Positive affect items | Sedikides et al., 2004 | Sedikides et al., 2004 | 3 |  | X |  |  |
| Positive Affect subscale of Subjective well-being Inventory | Zheng et al., 2001 | Ding et al., 2016 | 8 |  | X |  |  |
| Positive Affect subscale of Positive and Negative Affect Schedule (PANAS) | Watson et al., 1988 | Brunell & Buelow, 2019; Clark et al., 2010; Garcia et al., 2015; Giacomin & Jordan, 2016; Gornik-Durose, 2020; Hatchel et al., 2018; Konrath et al., 2014; Miller et al., 2010; Monacis et al., 2015; Morf et al., 2017; Naragon-Gainey & DeMarree, 2017; Rhodewalt et al., 1998; Sakkar Sudha & Shahnawaz, 2020; Tonna et al., 2018; Watson et al., 2005; Żemojtel‐Piotrowska et al., 2017; Yan, 2013 | 10 | Sample positive affect adjective descriptors include “excited” and “active.” | X |  |  |
| Positive Affect subscale of Positive and Negative Affect Schedule –Expanded Version (PANAS-X) | Watson & Clark, 1994 | Bogart et al., 2004 | 10 |  | X |  |  |
| Positive Subscale of Scale of Positive and Negative Experience (SPANE) | Diener et al., 2010 | Aminnuddin, 2020; Richardson et al., 2021; Zhang, 2011 |  | pleasant, happy | X |  |  |
| Positive Affect Subscale of Positive and Negative Affect Schedule for Children | Laurent et al., 1999 | Thomaes et al., 2010 |  | happy, active, proud | X |  |  |
| Single item measures of positive affect | Womick et al., 2019 | Womick et al., 2019 | 1 | “Rate the extent to which you feel happy” | X |  |  |
| Happiness Measures | Fordyce, 1988 | Chamberlain & Haaga, 2001 | 2 |  | X |  |  |
| Subjective Happiness Scale(SHS) | Lyubomirsky & Lepper, 1999 | Aghababaei & Błachnio, 2015; Brailovskaia & Margraf, 2016; Brailovskaia et al., 2019; Lopez, 2019; Thakur, 2018 | 4 | “Compared to most of my peers, I consider myself: 1 = *less happy*, 7 = *more happy* | X |  |  |
| Oxford Questionnaire of Happiness | Kołodziej et al., 2013 | Łojan, 2021 | 23 | “I’m very happy.” | X |  |  |
| School Children’s Happiness Inventory (SCHI) | Ivens, 2007 | Çevik et al., 2021 | 30 |  | X |  |  |
| Satisfaction with Life Scale (SWLS) | Diener et al., 1985 | Adeeb, 2020; Aghababaei & Błachnio, 2015; Akinci, 2015; Aminnuddin, 2020; Anderson & Costello, 2009; Ben Shlomo & Taubman-Ben-Ari, 2017; Błachnio & Przepiórka, 2018; Bourbonnais & Durand, 2018; Brailovskaia & Margraf, 2016; Brailovskaia et al., 2019; Chamberlain & Haaga, 2001; Edelstein et al., 2012; Fang et al., 2021; Giacomin & Jordan, 2016; Gornik-Durose, 2020; Górnik-Durose, 2021; Górnik-Durose & Pyszkowska, 2020; Hanke et al., 2019; Hernandez et al., 2021; Hill & Roberts, 2012; Horton, 2021; Hyun & Ku, 2021; Kaufman & Jauk, 2020; Limone et al., 2020; Liu et al., 2021; Lopez, 2019; Luo et al., 2020; Matshaba, 2018; Monacis et al., 2015; Morf et al., 2017; Musek & Grum, 2021; Ng et al., 2014; Richardson et al., 2021; Rohmann et al., 2019; Rose, 2002; Sakkar Sudha & Shahnawaz, 2020; Sedikides et al., 2004; Thakur, 2018; Thakur, 2018; Van Groningen et al., 2021; Volmer et al., 2019; Werner et al., 2019; Zajenkowski & Czarna, 2015; Zajenkowski et al., 2020b; Zajenkowski, 2021; Zappala, 2007; Zhang, 2011; Zhou et al., 2009; Zhu, 2016; Żemojtel‐Piotrowska et al., 2017 | 5 | “In most ways my life is close to my ideal.”  “I am satisfied with my life.” | X |  |  |
| Global life satisfaction | Beierlein et al., 2014 | Modersitzki et al., 2021 | 1 |  | X |  |  |
| Life satisfaction | Kaufman et al., 2020 | Kaufman et al., 2020 |  |  | X |  |  |
| Multidimensional Students’ Life Satisfaction Scale | Huebner, 1994; Tian & Liu, 2005 | Cai et al., 2017; Li et al., 2020 | 25 | “I like to be with my family.”  “My friends treat me well.” | X |  |  |
| General Life Satisfaction subscale of the Extended Satisfaction with Life Scales(ESWLS) | Alfonso et al., 1996 | Richardson et al., 2021 | 5 |  | X |  |  |
| Quality of Life Inventory (QOLI) | Frisch, 1994 | Aghababaei, 2014; Ellison et al., 2020 |  |  | X |  |  |
| Global Quality of Life | Hyland & Sodergren, 1996; Skevington et al., 2004 | Atroszko et al., 2018; Charzyńska et al., 2021; Huxley et al., 2021; Uzarska et al., 2023 | 1 | “How would you rate your quality of life?” | X |  |  |
| Riverside Life Satisfaction Scale (RLSS) | Margolis et al., 2018 | Sensoy, 2020 | 6 | “If I could live my life over, I would change many things.” (reverse-coded)  “I am satisfied with where I am in life right now.” | X |  |  |
| Hedonic Tone subscale of UWIST Mood Adjective Checklist (UMAC) | Matthews et al., 1990 | Zajenkowski & Czarna, 2015 | 29 |  | X |  |  |
| Life Well-Being subscale of the Employee Well-Being Scale | Zheng et al., 2015 | Meng et al., 2022 | 6 | “I feel satisfied with my life.” | X |  |  |
| Eudaimonic Well-Being Questionnaire | Waterman et al., 2010 | Werner et al., 2019 | 21 |  |  | X |  |
| Global Meaning in Life Questionnaire (GMLQ) | Costin & Vignoles, 2020 | Zhu et al., 2021 | 4 |  |  | X |  |
| Perceived Sacredness in Life Scale | Doehring et al., 2009 | Doehring et al., 2009 | 28 |  |  | X |  |
| Basic Psychological Needs Satisfaction Scale | Standage, 2005 | Cai et al., 2017 | 15 |  |  | X |  |
| Flourishing Scale | Diener et al., 2010 | Aminnuddin, 2020; Lopez, 2019 | 8 |  |  | X |  |
| Multidimensional Inventory of Religious/Spiritual Well-Being (RSWB) | Unterrainer et al., 2014 | Unterrainer et al., 2016 | 48 | “My faith gives me a feeling of security.” |  | X |  |
| Hope immanent subscale of Multidimensional Inventory of Religious/Spiritual Well-Being ( RSWB) | Unterrainer et al., 2014 | Unterrainer et al., 2016 |  | “I view the future with optimism.” |  | X |  |
| Sense and meaning subscale of Multidimensional Inventory of Religious/Spiritual Well-Being ( RSWB) | Unterrainer et al., 2014 | Unterrainer et al., 2016 |  | “I have experienced true (authentic) feelings.” |  | X |  |
| Framework Subscale of Life Regard Index (LRI) | Battista & Almond, 1973 | Zondag, 2005; Zondag et al., 2009 |  | “I have the feeling that I have found an important value worth striving for.” |  | X |  |
| Fulfilment Subscale of Life Regard Index (LRI) | Battista & Almond, 1973 | Zondag, 2005; Zondag et al., 2009 |  | “Life gives me a great deal of satisfaction.” |  | X |  |
| Lopez Meaning Scale (LMS) | Lopez, 2019 | Lopez, 2019 |  |  |  | X |  |
| Meaning in Life Questionnaire (MLQ) | Nelson et al., 2014 | Liu et al., 2021 | 10 | “I understand my life’s meaning.”  “I am always searching for something that makes my life feel significant.” |  | X |  |
| Presence of Meaning subscale from the Meaning in Life Questionnaire (MLQ-P) | Steger et al., 2006 | Horton, 2021; Lopez, 2019; Wang et al., 2019; Womick et al., 2019 |  | “I have a good sense of what makes my life meaningful.” |  | X |  |
| Meaningfulness and purpose of life | Prendergast et al., 2019 | Prendergast et al., 2019 | 1 | “Do you feel that your life is meaningful and has a purpose?” |  | X |  |
| Ryff's Scales of Psychological Well-being | Ryff, 1989 | Kaufman & Jauk, 2020; Thakur, 2018; Zappala, 2007 | 42 |  |  | X |  |
| Purpose in life subscale of Ryff's Scales of Psychological Well-being | Ryff, 1989 | Aghababaei & Błachnio, 2015; Kállay, 2020 | 7 |  |  | X |  |
| Shortened version of Ryff's psychological wellbeing measure | Ryff & Keyes, 1995 | Edelstein et al., 2012 | 18 | “I like most aspects of my  Personality.” |  | X |  |
| The Subjective Vitality Scale (SVS) | Ryan & Frederick, 1997 | Hewitt & Kealy, 2022 |  | “I feel alive and vital.”  “ I have energy and spirit.” |  | X |  |
| Religious Wellbeing subscale of the Spiritual Well-being Scale(SWB) | Ellison, 1983 | Horton et al., 2016 | 10 | “I have a personally meaningful relationship with God.” |  | X |  |
| Existential Wellbeing subscale of the Spiritual Well-being Scale (SWB) | Ellison, 1983 | Horton et al., 2016 |  |  |  | X |  |
| Seeking of Noetic Goals Test (SONG) | Crumbaugh, 1977 | Peebles, 2016 | 20 | “I think about the ultimate meaning of life …”  (rated from “*never*” to “*constantly*”) |  | X |  |
| The Purpose-in-Life Test (PIL) | Crumbaugh & Maholick, 1969 | Peebles, 2016 | 20 | “My personal existence is …  (rated from “*utterly meaningless and without purpose*” to “*very purposeful and meaningful*”) |  | X |  |
| Composite score of subjective wellbeing | Womick et al., 2020; Werner et al., 2019 | Womick et al., 2020; Werner et al., 2019 |  |  | X |  |  |
| Composite wellbeing score | Joshanloo, 2021; Womick et al., 2019; Zuckerman & O'Loughlin, 2009 | Joshanloo, 2021; Womick et al., 2019; Zuckerman & O'Loughlin, 2009 |  | “ I have energy and spirit.” |  |  | X |
| Subjective Well-Being Scale (SWBC) | Chen & Ji, 2006 | Tian et al., 2014 | 35 |  |  |  | X |
| Well-being subscale of Mental Health Test (MHT) | Vargha et al., 2020 | Grabovac & Kurbalija, 2021 | 3 | “In my everyday life there is significantly more happiness than sadness.” |  |  | X |
| Mental Health Continuum-Short Form (MHC-SF) | Keyes, 2002 | Gornik-Durose, 2020; Jankowski et al., 2022 | 14 | Hedonic (emotional—e.g. ‘‘How often did you feel happy?’’)  Eudaimonic (psychological—e.g. ‘‘How often did you feel good at managing the responsibilities of your daily life?’’ and social—e.g. ‘‘How often did you feel that you belonged to a community?’’) |  |  | X |
| General wellbeing schedule | Wang, 2008 | Wang, 2008 | 33 |  |  |  | X |
| Faces Scale (FS) | Andrews & Withey, 1976 | Rose, 2002 | 1 | The FS consists of seven faces that progress from a frowning face to a smiling face, and participants circled the face that best represented how they felt “about their life as a whole.” | X |  |  |
| General subjective well-being scale | Zhang, 2011 | Zhang, 2011 | 5 |  | X |  |  |
| Well-being Index (WHO-5) | Topp et al., 2015 | Quang et al., 2022 | 5 | “I have felt cheerful and in good spirits.” |  | X |  |

1. **CATEGORIZATION OF SELF-ESTEEM MEASURES**

| **Measures** | **Measure Reference** | **Included study** | **Number of items** | **Sample items** |
| --- | --- | --- | --- | --- |
| Rosenberg Self-esteem Scale (RSES) | Rosenberg, 1965 | Ng et al., 2014; Rose, 2002; Sedikides et al., 2004; Zuckerman & O'Loughlin, 2009; Wang, 2008 | 10 | “On the whole, I am satisfied with myself” |
| Single-item Self-esteem Scale (SISE). | Robins et al., 2001 | Rose, 2002 | 1 | “I have high self-esteem” |
| Fear of Inadequacy Scale (FIS) | Fleming & Courtney, 1984 | Sedikides et al., 2004 | 42 | “Do you ever think that you are a worthless individual?”. |
| Self-Liking / Self-Competence Scale (SLCS) | Tafarodi & Swann, 1995 | Sedikides et al., 2004 | 20 | Participants rated the frequency with which they experienced negative (unpleasant, sad, angry, afraid) and positive (happy, pleasant, joyful, contented) affect. |

1. **CORRELATIONS AMONG MODERATORS AND DESCRIPTIVE STATISTICS**

**Table 1S**

*Correlations and Descriptive Statistics of the Four Continuous Moderators (Hofstede Individualism, Age Mean, Study Year, Female Participants) and the Three Categorical Moderators (Wellbeing Forms, Publications Status, Study Design—Expressed as Percentage per Category)*

|  | *Mean/%* | *SD* | *1* | 2 | 3 | *4* |
| --- | --- | --- | --- | --- | --- | --- |
| 1. Hofstede Individualism | 69.09 | 29.51 | - |  |  |  |
| 2. Age Mean | 27.29 | 9.58 | 0.44^***^ | - |  |  |
| 3. Female Participants | 64.65 | 16.80 | 0.09 | 0.004 | - |  |
| 4. Study Year | 2015.78 | 5.57 | -0.23^***^ | -0.04 | -0.15^*^ | - |
| 5. Wellbeing Forms(%) |  | - | - | - | - | - |
| Eudaimonic | 17.04% | - | - | - | - | - |
| Hedonic | 78.48% | - | - | - | - | - |
| Mixed | 4.48% | - | - | - | - | - |
| 6. Publication Status (%) |  | - | - | - | - | - |
| Unpublished | 8.52% | - | - | - | - | - |
| Published | 91.48% | - | - | - | - | - |
| 7. Study Design (%) |  | - | - | - | - | - |
| Cross-Sectional Study | 91.48% | - | - | - | - | - |
| Longitudinal Study | 8.52% | - | - | - | - | - |

1. **BIVARITE MODERATORS ANALYSES**

**Table 2S**

*Bivariate Moderator Analyses with Pairwise Comparison*

| Correlation | Difference in *β* | | *SE* | *t* | 95% CI | *p* |
| --- | --- | --- | --- | --- | --- | --- |
| **Association Between Grandiose Narcissism and Wellbeing** | |  |  |  |  |  |
| Hedonic vs. Eudaimonic | | 0.01 | 0.03 | 0.47 | [-0.04, 0.07] | .642 |
| Mixed vs. Eudaimonic | | 0.09 | 0.06 | 1.55 | [-0.02, 0.20] | .121 |
| Mixed vs. Hedonic | | 0.07 | 0.05 | 1.43 | [-0.03, 0.18] | .154 |
| **Association Between Vulnerable Narcissism and Wellbeing** | |  |  |  |  |  |
| Hedonic vs. Eudaimonic | | 0.07 | 0.04 | 1.51 | [-0.02, 0.16] | .137 |
| Mixed vs. Eudaimonic | | 0.04 | 0.08 | 0.43 | [-0.13, 0.20] | .667 |
| Mixed vs. Hedonic | | -0.03 | 0.08 | -0.43 | [-0.18, 0.12] | .669 |

1. **SENSITIVITY ANALYSIS WINSORIZING OUTLIERS**

We identified two large negative effect sizes (*r* = -.36, -.40) as potential outliers in the association between grandiose narcissism and wellbeing. In line with previous research (Orth et al., 2021) and methodological guidelines (Viechtbauer & Cheung, 2010), we chose to include all effect sizes in the analyses reported in our article. Nevertheless, we conducted an additional sensitivity analysis to investigate the potential influence of outliers in our analyses. To avoid dropping effect sizes that truly are extreme in size because of sampling variation, we adjusted the outliers with a new value that has a *Z* score of -3.29. ﻿After adjusting the outliers, the overall effect size remained unchanged, *r* = .19, 95% CI [.16, .21], *p* < .001. We found a heterogeneous distribution of effect sizes, both within studies (i.e., variance at level 2), χ^2^(1) = 173.47, *p* < .001, and between studies (i.e., variance at level 3), χ^2^(1) = 19.00, *p* < .001. Thus, we proceeded with moderation analyses. All such analyses results remained largely similar. Individualism moderated the association between grandiose narcissism and wellbeing, *F*(1, 216) = 4.21, *p* = .011. This association was stronger in samples from countries with higher (than lower) levels of individualism, β_1_ = 0.001, 95% CI = [0.000, 0.002]. The confidence interval does not include zero. All the other sample and study characteristics did not significantly moderate the association between grandiose narcissism and wellbeing.

Then we explored whether the association between narcissism and wellbeing varied across effect size type (i.e., zero-order vs. controlling for self-esteem) after adjusting the outliers. The effect size type (i.e., zero-order vs. controlling for self-esteem) moderated the association between grandiose narcissism and wellbeing, *F*(1, 240) = 23.15, *p* < .001, such that this association was not significant when controlling for self-esteem, *r* = .01, 95% CI = [-.07, .08], *p* = .867, but was significant when not controlling for self-esteem, *r* = .19, 95% CI = [.16, .22], *p* < .001.

We also tested the interaction between individualism and narcissism form on the full dataset after adjusting the outliers. This interaction was still significant, β_1_= 0.002, 95% CI [0.001, 0.004], *p* = .010, indicating that the discrepancy between the two narcissism forms’ associations with wellbeing enlarges as individualism increases.
